# The cuproptosis-related signature predicts the prognosis and immune microenvironments of primary diffuse gliomas: a comprehensive analysis

**DOI:** 10.1186/s40246-024-00636-2

**Published:** 2024-07-02

**Authors:** Tao Chang, Yihan Wu, Xiaodong Niu, Zhiwei Guo, Jiahao Gan, Xiang Wang, Yanhui Liu, Qi Pan, Qing Mao, Yuan Yang

**Affiliations:** 1 Department of Neurosurgery, West China Hospital, Sichuan University, No. 37 Guo Xue Xiang, Chengdu, 610041 China; 2 National Clinical Research Center for Geriatrics, West China Hospital, Sichuan University, Chengdu, 610041 China; 3 School of Clinical Medicine, Jiangxi University of Traditional Chinese Medicine, Nanchang, 330004 China; 4 Department of Dermatology, Chongqing Traditional Chinese Medicine Hospital, Chongqing, 400013 China

**Keywords:** Cuproptosis, Cuproptosis-related genes, Immune microenvironment, Prognosis, Glioma

## Abstract

**Background:**

Evidence has revealed a connection between cuproptosis and the inhibition of tumor angiogenesis. While the efficacy of a model based on cuproptosis-related genes (CRGs) in predicting the prognosis of peripheral organ tumors has been demonstrated, the impact of CRGs on the prognosis and the immunological landscape of gliomas remains unexplored.

**Methods:**

We screened CRGs to construct a novel scoring tool and developed a prognostic model for gliomas within the various cohorts. Afterward, a comprehensive exploration of the relationship between the CRG risk signature and the immunological landscape of gliomas was undertaken from multiple perspectives.

**Results:**

Five genes (*NLRP3*, *ATP7B*, *SLC31A1*, *FDX1*, and *GCSH*) were identified to build a CRG scoring system. The nomogram, based on CRG risk and other signatures, demonstrated a superior predictive performance (AUC of 0.89, 0.92, and 0.93 at 1, 2, and 3 years, respectively) in the training cohort. Furthermore, the CRG score was closely associated with various aspects of the immune landscape in gliomas, including immune cell infiltration, tumor mutations, tumor immune dysfunction and exclusion, immune checkpoints, cytotoxic T lymphocyte and immune exhaustion-related markers, as well as cancer signaling pathway biomarkers and cytokines.

**Conclusion:**

The CRG risk signature may serve as a robust biomarker for predicting the prognosis and the potential viability of immunotherapy responses. Moreover, the key candidate CRGs might be promising targets to explore the underlying biological background and novel therapeutic interventions in gliomas.

**Supplementary Information:**

The online version contains supplementary material available at 10.1186/s40246-024-00636-2.

## Introduction

Gliomas, characterized by infiltrative growth, are the most prevalent primary intracranial tumors of the central nervous system in adults, with an incidence of 5–6/100,000 each year [1–5]. In 50% of cases, glioblastoma (GBM) is extremely deadly, with its 5-year survival rate not exceeding 7.2% [6]. Conventional therapies for GBM including surgery, chemotherapy, and radiotherapy, have only marginally improved patient outcomes, extending overall survival by just 4.9 months [7, 8]. Hence, there is an urgent need to explore innovative therapies for glioma patients.

Immunotherapy has emerged as a promising strategy for glioma treatment, leveraging the interaction between the brain's lymphatic vessels and the external immune system [9]. Various types of immunotherapy, such as adoptive cell transfer and immune checkpoint inhibitors (ICIs), have demonstrated significant clinical responses, but their efficacy varies among different cancer subsets [10, 11]. While the focus is primarily on immune checkpoints like the programmed cell death protein 1 (PD1) and cytotoxic T lymphocyte-associated antigen 4 (CTLA4), scarce clinical trials conducted on ICIs for gliomas have yielded significant outcomes [12–14]. Tumor-infiltrating immune cells and other distinct tumor microenvironment (TME) components foster an immunosuppressive milieu, enabling cancer cells to evade immune system surveillance [15]. Furthermore, the heterogeneous nature of tumor antigens within subtypes makes it challenging to determine effective immunotherapy options [16]. These factors collectively contribute to the limited success of immunotherapy in gliomas.

Given the pivotal role of copper in cellular signaling, it may implicate the development and progression of carcinogenesis, particularly through mechanisms such as cell proliferation, angiogenesis, and metastasis [17]. Cuproptosis, a novel form of programmed cell death distinct from apoptosis, ferroptosis, pyroptosis, and necroptosis [18], remains relatively unexplored in terms of its impact on tumor cells. Nineteen cuproptosis-related genes (CRGs) have been reported [19], involved in essential functions such as copper ion homeostasis, protein lipoylation, the tricarboxylic acid cycle, and oxidative stress responses [18]. Previous researches have demonstrated the beneficial effects of a model constructed based on CRGs for predicting the prognosis of breast, and neck squamous cell carcinoma [19, 20]. With the targeting of cuproptosis considered as a potential option for tumor management, the impact of CRGs on the prognosis and the immunological landscape of gliomas is yet to be fully understood.

To determine the impact of cuproptosis on the TME immune features in gliomas, we explored the CRG expression patterns and developed a prognostic model across multiple cohorts. Additionally, we explored the correlation between the CRG risk signature and the immunological landscape of gliomas from various perspectives.

## Materials and methods

### Data collection

We sourced glioma RNA sequencing profiles and clinical records from The Cancer Genome Atlas (TCGA database, https://portal.gdc.cancer.gov/) and Chinese Glioma Genome Atlas (CGGA) curation (http://www.cgga.org.cn/.), comprising 615 primary gliomas from TCGA and 406 cases from CGGA. Additionally, 85 patients were enrolled from the in-house Sichuan West China Hospital (SWCH) cohort, the sequencing data of which can be obtained at the Open Archive (https://ngdc.cncb.ac.cn/omix/). All patients met the following inclusion criteria: supratentorial lesions, primary diagnosis of glioma, adults ≥ 18 years, and a definitive pathologic diagnosis, mRNA sequencing profile, and complete clinical phenotypes.

### Construction of CRG and immune scores

In the TCGA datasets, CRGs were screened using the least absolute shrinkage and selection operator (LASSO) and Cox regression employing the “glmnet” R package (R Foundation, Vienna, Austria, version 4.1.2). These CRGs, whose coefficients at the lambda minimum C-index were not 0, were identified as potential genes. Subsequently, five potential target genes (*NLRP3*, *ATP7B*, *SLC31A1*, *FDX1*, and *GCSH*) for the model were analyzed using the "survminer" R package to perform a multivariate Cox regression analysis. Then, these genes were used to develop a prognostic signature. The score was calculated using the following formula:

1 $$\text{CRG score }= \sum\limits_{i=1}({\beta }_{i} * {Exp}_{i})$$

where *β* and *Exp* are the coefficients and expression of each critical gene, respectively.

A gene expression profile was extracted to calculate the stromal, immune, and estimate scores, along with tumor purity using the “ESTIMATE” package in R [21]. Then, patients were stratified into high- and low-risk subgroups according to the median immune and CRG scores, respectively.

### Identification of Differentially Expressed Genes (DEGs) and functional annotation

Using the R package “limma”, differentially expressed genes (DEGs) were identified by separately comparing the immune and CRGs subgroups. The significance of the DEGs was determined by the false discovery rate < 0.05 and a cutoff of |Log2 Fold Change (FC)|> 1. An adjusted *p*-value < 0.05 indicated statistical significance between the different subgroups.

The 'clusterProfiler' R package was used to analyze the enrichment of differentially expressed genes (DEGs) in the Gene Ontology (GO) and Kyoto Encyclopedia of Genes and Genomes (KEGG) databases, with the top 10 enrichment terms displayed [22, 23]. Dot plots and enriched KEGG pathways were employed to explore biological functions and signaling pathways, with significantly enriched terms meeting the criteria of *p* value < 0.05 and *q* value < 0.05. Subsequently, gene set enrichment analysis (GSEA) was performed using the 'gsva' R package to identify significant alterations in signaling pathways for each subtype [24]. The results were visualized using 'gseaplot2' to display the top five subgroup enrichment terms.

### Nomogram construction and evaluation

A univariate Cox regression analysis was performed to analyze clinical indicators, CRG and immune scores, and other potential prognostic factors. Next, a multivariate Cox regression analysis was conducted for variables with *p* values < 0.1 in the univariate analysis. The *p* value, hazard ratio, and 95% confidence interval of each variable were then displayed using the “forestplot” R package. An overall score based on the nomogram model was obtained by summing the scores for each clinically significant factor. Additionally, calibration plots were generated to assess the predictive accuracy for 1-, 2-, and 3-year prognosis compared to virtually observed outcomes. A decision curve analysis (DCA) using the R package “ggDCA” was performed to determine the net benefit of using the model at different threshold probabilities. Finally, receiver operating characteristic (ROC) curves were constructed with the R package “rms” to evaluate the effectiveness of the nomogram in predicting prognosis for patients with gliomas.

### Estimation of Tumor-infiltrating Immune Cells, Tumor Mutant Burden (TMB), Tumor Immune Dysfunction and Exclusion (TIDE), Specific Markers for Cytotoxic T Lymphocytes (CTLs) and immune exhaustion, immune checkpoints, and cytokines in gliomas

Due to the challenge of determining the specific percentage of immune cells based on the immune scores, the “CIBERSORT” package was utilized to estimate the composition of 22 tumor-infiltrating immune cells [25].

The somatic mutation files were retrieved from the TCGA database in the Varscan file format, while copy number variation (CNV) data were downloaded from UCSC Xena (https://xenabrowser.net/datapages/). A total of 598 patients in the TCGA cohort were eligible for TMB analysis, where the R package "maftool" was used to calculate significantly mutated genes and TMB [26].

T-cell exclusion and microsatellite instability analysis in the TME for ICIs response prediction were conducted using the TIDE site (http://tide.dfci.harvard.edu).

Finally, we separately examined the relationship between CRGs and molecular markers specific to cytotoxic T lymphocytes (CTLs), immune exhaustion, immune checkpoints of tumor signature genes, and cytokines in gliomas.

### Statistical analyses

All data analyses were conducted using R software (R Foundation, Vienna, Austria, version 4.1.2). Categorical data were analyzed using Chi-square or Fisher’s exact tests. Differences between the Kaplan–Meier curves were determined by the log-rank test. Missing values were imputed through a random forest algorithm that dynamically adjusted parameters to improve data accuracy, and statistical analysis was not performed on variables with missing values exceeding 5%. A *p* value < 0.05 was considered statistically significant.

## Results

### Construction of the CRG scoring system

The clinicopathological records of the three cohorts were comprehensively analyzed and are presented in Additional file 1: Fig. S1. Remarkable differences in age, WHO grade, molecular diagnosis, radiotherapy, TMZ therapy, and prognosis for gliomas were observed among the three datasets.

**Fig. 1:**
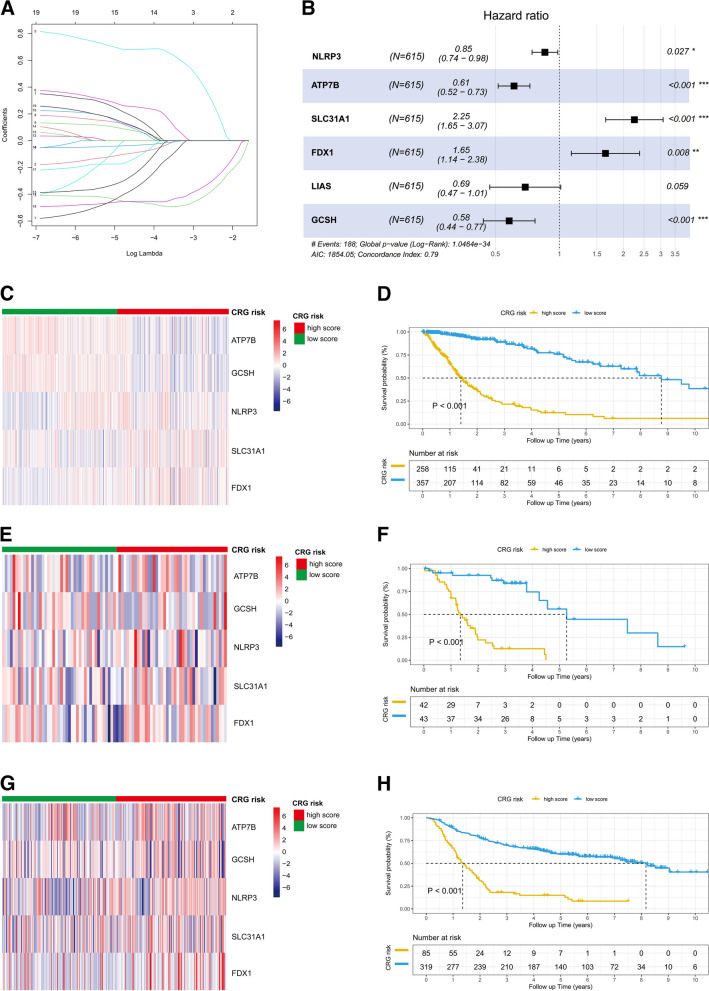
Development of the CRG prognostic signature in three cohorts. **A** Ten-times cross-validation for CRGs selection in the LASSO Cox regression. **B** Multivariate analyses in the TCGA cohort. **C** Heatmap of the expression of five CRGs between CRG risk subgroups. **D** Kaplan–Meier curves for CRG risk subgroups in the TCGA cohort. The heatmap of five CRG genes and Kaplan–Meier curves between CRG subgroups in the SWCH (**E**, **F**) and CGGA cohorts (**G**, **H**), respectively. *p < *0.05*; **p < *0.01*; ***p < *0.001*

In the TCGA cohort, relationships between the expression of 19 CRGs and clinicopathological features are shown in Additional file 2: Fig. S2A and B. Following multivariate Cox regression analysis and LASSO Cox regression, we identified *NLRP3*, ATP7B, *SLC31A1*, *FDX1*, and *GCSH* as potentially critical genes for constructing the prognostic signature in gliomas (Fig. 1A and B). Notably, SLC31A1 exhibited the highest expression levels among these genes, and significant differences in survival were noted for these five key CRGs (Additional file 2: Fig. S2C-G). The formula for the CRG score was as follows:

2 $$\mathrm{CRG}\;\mathrm{score}\hspace{0.17em}=\hspace{0.17em}(-0.157\ast NLRP3\;\mathrm{expression})\hspace{0.17em}+\hspace{0.17em}(-0.492\ast ATP7B\;\mathrm{expression})\hspace{0.17em}+\hspace{0.17em}(0.812\ast SLC31A1\;\mathrm{expression})\hspace{0.17em}+\hspace{0.17em}(0.500\ast FDX1\;\mathrm{expression})\hspace{0.17em}+\hspace{0.17em}(-0.544\ast GCSH\;\mathrm{expression}).$$

Afterward, patients were stratified into high- and low-risk subgroups according to the median CRG score. In the CRG high-score group, *ATP7B*, *NLRP3*, and *GCSH* had significantly lower expression, while *SLC31A1* and *FDX1* exhibited higher expression (Fig. 1C). Survival analysis indicated that patients in the high-score group revealed an inferior prognosis compared to those in the low-score group (Fig. 1D). Consistent with findings from the TCGA cohort, the expression patterns of these five genes and the Kaplan–Meier survival curves between CRG risk subgroups were also analyzed for the SWCH (Fig. 1E and F) and CGGA cohorts (Fig. 1G and H).

### Functional annotation in distinct risk subgroups

**Fig. 2:**
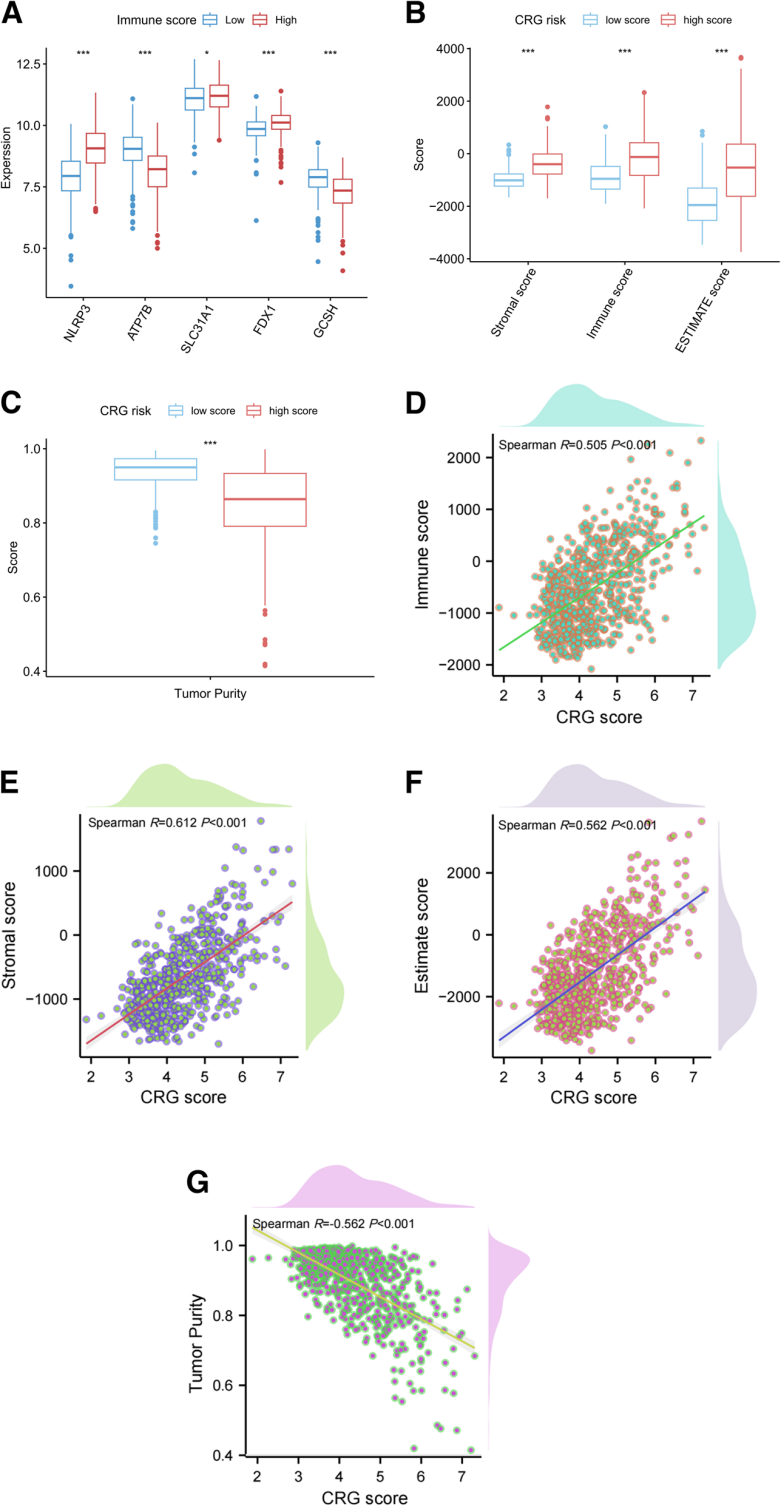
Relationship between immune scoring system and CRG prognostic signature. **A** Relationship between the immune score and expression of five CRGs. Relationship between immune scoring system (**B**) and CRG risk subgroups (**C**). **D-G** Linear regression between the CRG and immune scoring system. *p < *0.05*; **p < *0.01*; ***p < *0.001*

Regarding the correlation between CRG and immune scoring systems, significant statistical differences were observed in the expression of these five CRGs across immune risk subgroups. Specifically, *ATP7B* and *GCSH* were characterized by lower expression, while *NLRP3*, *SLC31A1*, and *FDX1* exhibited higher expression in the immune high-score group (Fig. 2A). Further, high CRG subgroups were associated with elevated immune, stromal, and estimated scores, alongside reduced tumor purity (Fig. 2B and C). Notably, the CRG score demonstrated a positive correlation with the immune, stromal, and estimate scores, but an inverse relationship with tumor purity (Fig. 2D-G).

We identified 999 intersecting DEGs in CRG and immune risk subgroups (Additional file 3: Fig. S3A) and specifically extracted the top 30 upregulated and downregulated genes respectively in the TCGA cohort. Additionally, there were some intersecting genes among the top 30 upregulated and downregulated DEGs between the immune and CRG risk groups (Additional file 3: Fig. S3B and C).

**Fig. 3:**
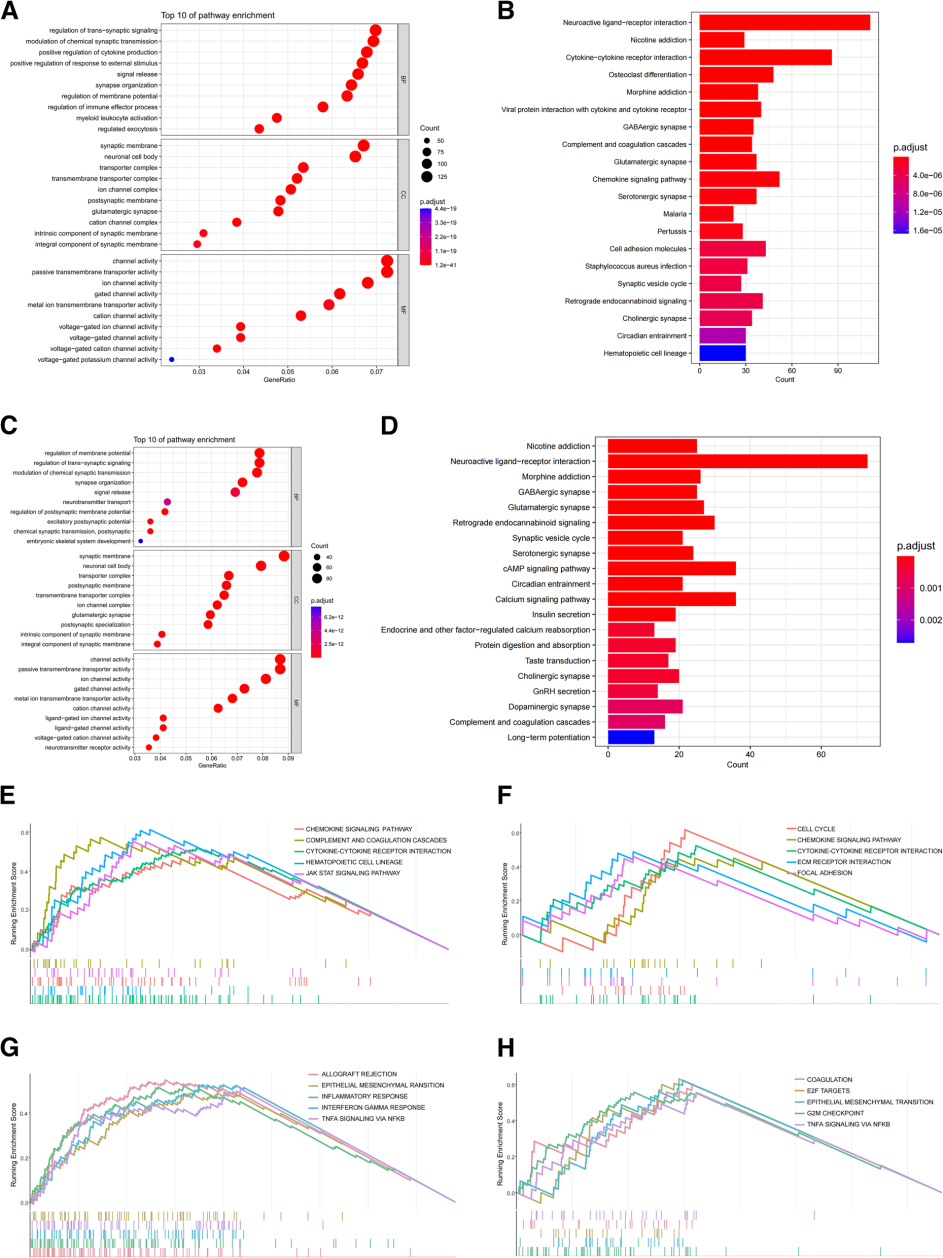
Functional enrichment analysis. GO enrichment analysis of the top 10 pathways (**A**), and KEGG enrichment analysis of the top 20 pathways (**B**) for distinct immune risk groups. **C **and** D** GO and KEGG in CRG risk subgroups. The top five pathways with the highest NES in KEGG (**E, F**) and HALLMARK (**G, H**) gene sets for the immune and CRG subgroups, respectively

A GO analysis revealed that DEGs in the immune subgroups were primarily involved in immune response-related pathways, such as cytokine production, immune effector processes, and myeloid leukocyte activation (Fig. 3A). The analysis also revealed high enrichment in cytokine receptor interactions, chemokine signaling pathways, and cell adhesion molecules in KEGG for the immune risk groups (Fig. 3B). Additionally, GO and KEGG annotations for the CRG subgroups were primarily related to the regulation of membrane potential, regulation of trans-synaptic signaling, modulation of chemical synaptic transmission, and cell signaling biological processes, including neuroactive ligand-receptor interaction and the cAMP signaling pathway, respectively (Fig. 3C and D). For the immune and CRG subgroups, some common mechanisms, including chemokine signaling, and cytokine receptor interactions, were observed in the top five signaling pathways of KEGG gene set (Fig. 3E and F), while epithelial-mesenchymal transition and TNF-α signaling via NF-κB were noted in the top five signaling pathways of HALLMARK gene set (Fig. 3G and H). Similarly, some of these, such as the P53 signaling pathway, ECM receptor interaction, chemokine signaling in the KEGG gene set, and TNFα signaling by the NF-kB, epithelial-mesenchymal transition, and inflammatory response in the HALLMARK gene set (Additional file 3: Fig. S3D and E), were associated with immunological activation. These findings underscore that CRGs may exert anti-tumor effects through immunoregulatory mechanisms.

### Nomogram for predicting glioma prognosis

**Fig. 4:**
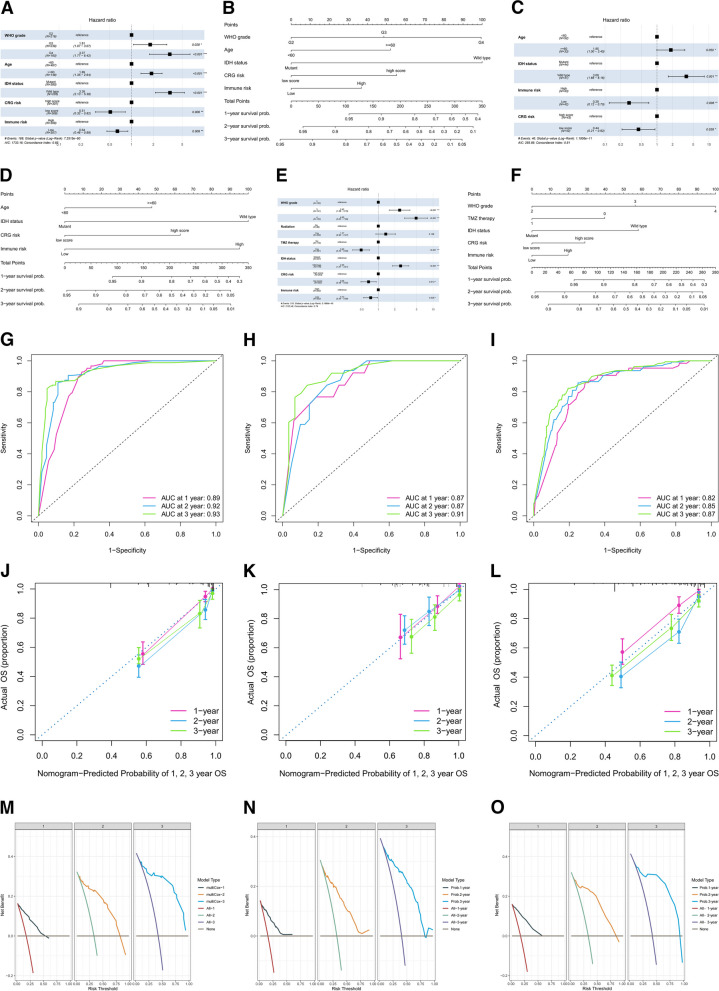
Nomogram construction and evaluation. Multivariate Cox regression of potential prognostic power in the TCGA (**A**), SWCH (**C**), and CGGA (**E**) cohorts. The nomogram was used to calculate the 1-, 2-, and 3-year prognosis for patients with gliomas in the TCGA (**B**), SWCH (**D**), and CGGA (**F**) cohort. ROC curves for predicting survival in the TCGA (**G**), SWCH (**H**), and CGGA (**I**) cohort. Calibration curve and decision curve evaluation for nomograms in the TCGA (**J**,** M**) cohort, SWCA (**K**,** N**) cohort, and CGGA (**L**, **O**) cohort. *p < *0.05*; **p < *0.01*; ***p < *0.001*

We developed a model to predict the survival for patients with gliomas at 1, 2, and 3 years in the three datasets. Within the TCGA cohort, factors including WHO grade, age, IDH status, CRG risk, and immune risk were identified as independent prognostic factors (*p* < 0.05) (Fig. 4A). These variables were integrated to construct the nomogram for individualized prognosis prediction (Fig. 4B). The ROC curve indicated that the nomogram had a superior predictive performance (AUC of 0.89, 0.92, and 0.93 at 1, 2, and 3 years, respectively) (Fig. 4G). A validation study was performed in the SWCH (Fig. 4C, D and H) and CGGA cohorts (Fig. 4E, F and I). Finally, calibration curves and decision curve analysis validated the performance of this model in predicting the prognosis of patients with gliomas, with high consistency between the actual proportion of the 1-, 2-, and 3-year overall survival and the nomogram-predicted probability in the three cohorts (Fig. 4J-O).

### Correlation between CRG Risk, and immune cell profiles in gliomas

**Fig. 5:**
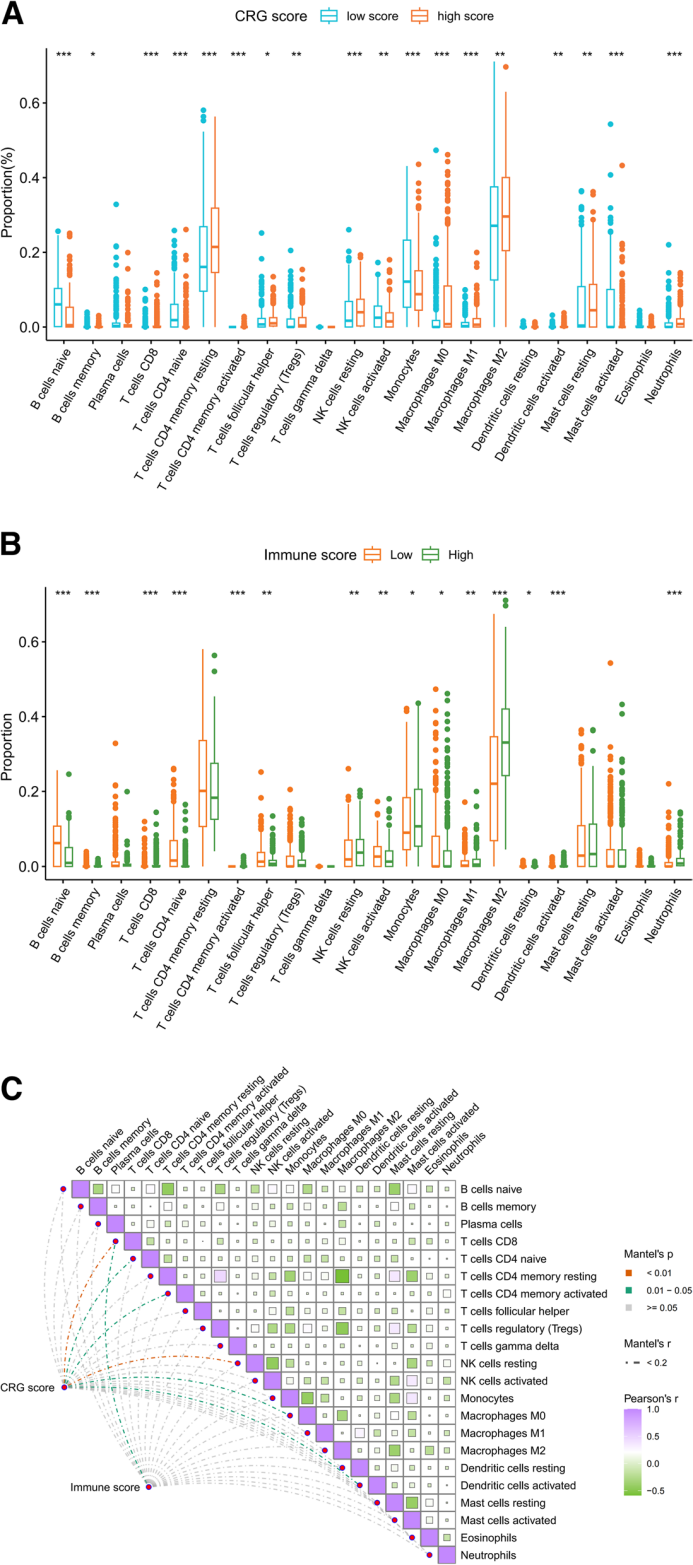
Immune cell profiles in gliomas. Infiltrating levels of 22 immune cells in CRG (**A**) and immune (**B**) risk subgroups. **C** Correlation between the 22 types of immune cells, CRG, and immune score. *p < *0.05*; **p < *0.01*; ***p < *0.001*

We used the CIBERSORT method to analyze 22 types of immune cell profiles. M2 macrophages (30%), and resting memory CD4 + T cells (20%) were the predominant infiltrating immune cells (Additional file 4: Fig. S4A and B). These two types of immune cells had more infiltration in the CRG and immune high-risk subgroups, although no statistically significant difference was observed in resting memory CD4 + T-cell infiltration between the immune subgroups (Fig. 5A and B) (Additional file 4: Fig. S4C). Additionally, a correlation was observed between the CRG score and infiltration of CD8 + T cells, naïve CD4 + T cells, activated memory CD4 + T cells, resting NK cells, M0 macrophages, and activated dendritic cells; however, only the correlation with CD8 + T cells was validated by the immune score (Fig. 5C), suggesting that the CRG score may be more closely related to immune cell infiltration in gliomas than the immune score.

### Analysis of genetic mutations, TIDE, Cytotoxic T Lymphocytes (CTLs) and immune exhaustion related molecular markers, immune checkpoints and cytokines in gliomas

**Fig. 6:**
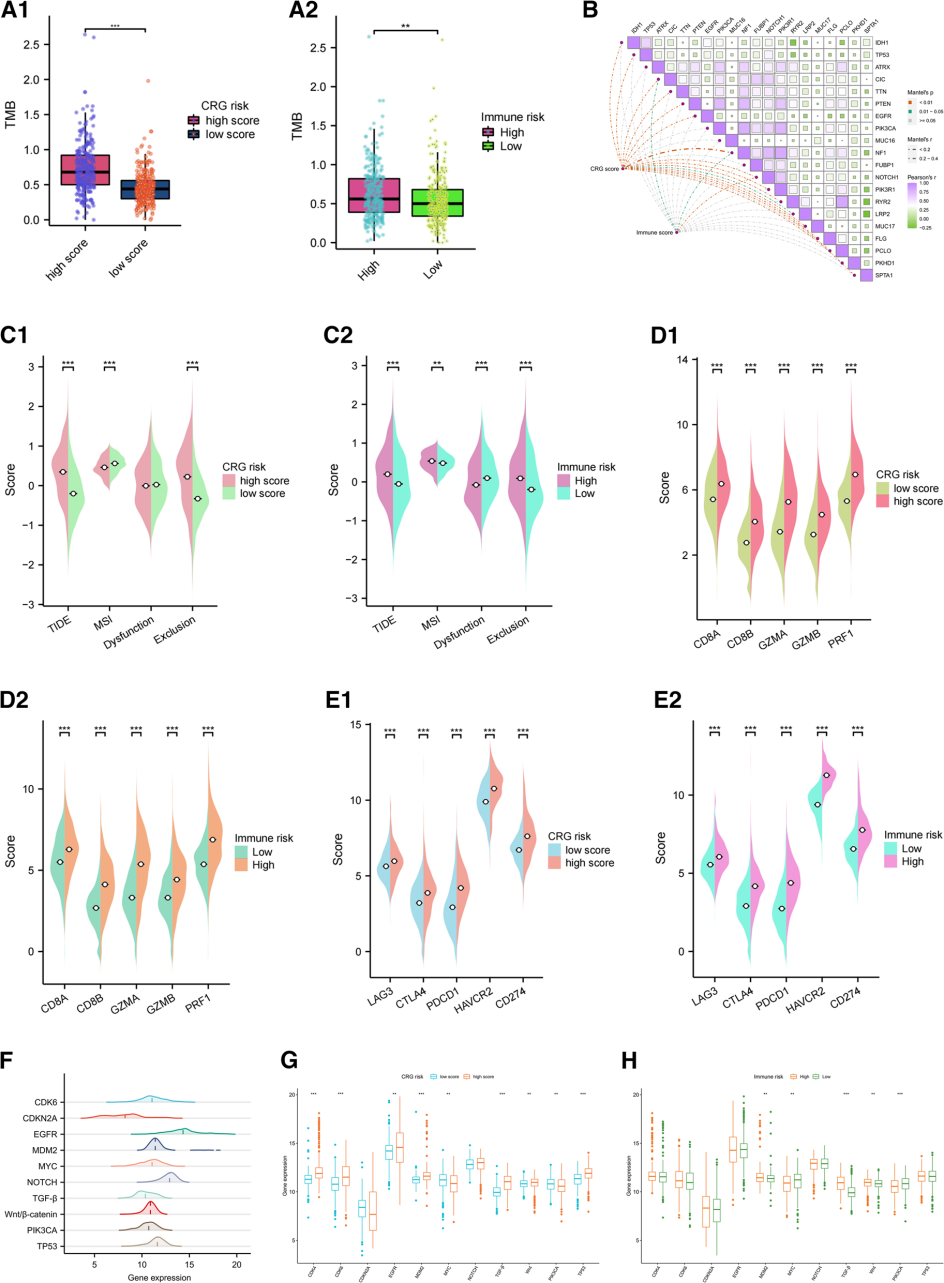
Molecular phenotypes of immune microenvironments in gliomas. **A** Relationship between TMB, CRG and immune risk groups. **B** Relationship between mutated genes, CRG, and immune scores. **C** Analysis of correlation between TIDE, and exclusion scores with the CRG and immune high-risk groups. The expression of cytotoxic T lymphocyte markers (**D**), and immune exhaustion-related genes (**E**) were all significantly higher in the CRG and immune high-risk groups. **F** Expression levels of 11 well-known biomarkers of cancerous signaling pathways. Pathway markers were differentially expressed in the CRG (**G**) and immune (**H**) high-risk subgroups. *p < *0.05*; **p < *0.01*; ***p < *0.001*

TMB, which indicates the frequency of genetic mutations in cancer and the potential to recruit more neoantigens, was positively correlated with the CRG score (Additional file 5: Fig. S5A). Additionally, TMB was significantly higher in both the CRG and immune high-score groups (Fig. 6A). *IDH1* was the most mutated gene (58%; with missense mutations) (Additional file 5: Fig. S5B). Notably, more mutated genes were associated with the CRG score than with the immune score, and *NF1* exhibited the strongest correlation among the top 20 mutated genes (Fig. 6B).

TIDE algorithms were used to examine T-cell dysfunction and exclusion scores, which could account for the differences in immune responses between the immune and matrix components. A positive correlation was noted between TIDE, and exclusion scores with CRG score, and this correlation was also observed in the immune score. Interestingly, microsatellite instability (MSI) showed a negative correlation with the CRG score but a positive correlation with the immune score (Additional file 5: Fig. S5C-J). Moreover, higher TIDE and exclusion scores were significant in the high-risk CRG and immune subgroups, while the MSI score did not show a consistent trend (Fig. 6C).

The CTL-related genes (*CD8A*, *CD8B*, *GZMA*, *GZMB*, and *PRF1*) (Fig. 6D), as well as the immune exhaustion-related genes (*LAG3*, *CTLA4*, *CD274*, *PD*-*L1*, and *HAVCR2*) (Fig. 6E), were all highly expressed in the CRG and immune high-score groups. The analysis of 11 biomarkers associated with well-known cancer signaling pathways revealed that *CDKN2A* was characterized by the lowest abundance, while EGFR exhibited the highest expression (Fig. 6F). Additionally, the pathway markers (*PIK3CA, Wnt, TGF-β, MYC,* and *MDM2*) showed differential expression in the immune risk subgroups as well as between CRG risk subgroups (Fig. 6G and H).

Among the 51 immune checkpoints, *SIRPα* and *CD47* were highly expressed, with discernible differences among the CRG risk subgroups. In particular, low expression of these two markers correlated with a high CRG score and worse prognosis (Additional file 5: Fig. S5K and L).

Cytokines play a crucial role in information transmission between cells and regulation of the TME. The majority of cytokines exhibited higher expression in patients with high CRG scores (Additional file 6: Fig. S6A), and most were more closely associated with the CRG score than the immune score (Additional file 6: Fig. S6B).

## Discussion

This seminal research involved the development and validation of a CRG risk signature to predict the prognosis of glioma patients. Compared to similar studies [27–29], the model featuring the CRG risk signature displayed exceptional stability as an independent prognostic factor across various cohorts, even exceeding 90% for 3-year survival predictions.

The correlation between immune scores calculated by the ESTIMATE algorithm and the clinicopathologic features, as well as the TME, has been comprehensively identified across various types of cancers [21, 30]. A striking observation was that, unlike tumors in peripheral organs, where high immune scores typically indicate a better prognosis [30, 31], glioma patients with high CRG and immune risk scores had worse outcomes, possibly due to the distinct immune microenvironment of gliomas.

Gliomas, characterized by distinct TME features, generally lack abundant activated CD8 + T and NK cells, which are considered as indicators of immune recognition and representative of "hot" tumors [32–34]. Despite a uniquely high ratio of activated CD8 + T cells in the CRG high-risk subgroups, their absolute content was markedly low, posing challenges in inducing direct tumoricidal potential. The high-risk subgroups also exhibited significant enrichment in immunosuppressive properties of cells, including M2 macrophages comprising about 30%, which play a crucial role in secreting chemokines and upregulating immunosuppressive proteins to dampen T cell functionality [35, 36]. Moreover, the higher enrichment of resting memory CD4 + T cells in the CRG and immune high-risk subgroups implies that their activation, proliferation, and differentiation into specific Th subsets may offer new insights into the pathogenesis and therapy for gliomas [37].

The exhaustion of CD8 + T and NK cells, along with the overexpression of molecular markers such as LAG3, CTLA4, CD274, PD-L1, and HAVCR2, have been identified as key mechanisms by which cancer cells can escape host immunity [38, 39]. Conversely, immune activity-related signatures, including PRF1, GNLY, GZMA, GZMB, and interferon regulatory factor 1, play crucial roles in tumor cell recognition and tumoricidal, with connections to extended survival, and identifying responders to anti-PD-1 antibody treatment [40, 41]. Interestingly, the increased expression of both immune activity and immune exhaustion-related molecules associated with the high CRG scores was indicative of an unfavorable prognosis, possibly due to the varying expression levels of these biomarkers. For instance, HAVCR2, an immune exhaustion molecule, exhibited the highest expression, potentially triggering significant immune evasion in glioma cells [42]. Meanwhile, TIDE and exclusion scores showed a positive correlation with the CRG score, while MSI exhibited a negative correlation with these phenotypes. These findings further signified that glioma cells may possess a greater immune evasion potential through T-cell exclusion, making it less likely for patients with these phenotypes to benefit from immunotherapy [43].

Although certain immune checkpoints, including CD274 (PD-L1), PDCD1 (PD-1), and CTLA-4, have been effectively employed in targeted immunotherapy for other organ tumors [44], this study explored that the expression of these molecules was notably low in gliomas. In contrast, critical biomarkers for glioblastoma, including CD47 and SIRPα, exhibited higher expression among these immune checkpoints, which are correlated with a CRG low-risk signature and improved prognosis. SIRPα, found on myeloid cells like macrophages, dendritic cells, neutrophils, neurons, and microglia, binds to CD47 with high affinity [45, 46]. Targeting the CD47-SIRPα immune checkpoints has emerged as a promising strategy in both preclinical and clinical studies, with the potential to modify the tumor microenvironment, restore innate and adaptive immune functions, and enhance the prognosis of gliomas [47].

A broad panel of chemokines and cytokines within the TME constitute a dynamic network that regulates the overall composition of immune cells infiltrating the tumor. The lack of T-cell-recruiting signals, such as chemokines directing T-cell trafficking (e.g., CXCL9, CXCL10, CXCL11, CXCL13, CCL2, and CCL5), could be a primary cause of T-cell exclusion [36, 48, 49]. EGFR, the most prevalent molecule in the study, exhibited differential expression in CRG subgroups, contributing to tumor cell differentiation, proliferation, and migration by regulating several signaling pathways, including PI3K/AKT, RAS/MAPK, and JAK2/STAT [50, 51]. Thus, further investigation into the relationship between cuproptosis and EGFR may shed new light on the application of EGFR inhibitors in gliomas [52].

Although this study provides important evidence to explore the potential of CRGs as markers for evaluating the TME, immunotherapy response and prognosis in gliomas, our findings should be interpreted with the following limitations in mind: First, as a retrospective study based on three independent datasets, glioma tissue mRNA sequencing profiles and clinical records are subject to selection bias. Second, while only transcriptional expression was considered in the molecular stratifications, it is essential to include metabolic, proteomic, and imaging characteristics. Third, in-vivo and in-vitro wet lab experiments should be conducted to verify the roles of CRG regulatory molecules in gliomas. In this regard, we intend to conduct such experiments in future work.

## Conclusions

It is conceivable that CRG risk signature may be a potent biomarker for predicting the prognosis and the potential viability of immunotherapy responses in gliomas. Moreover, the key candidate CRGs might be promising targets to explore the underlying biological background and novel therapeutic interventions in gliomas.

### Supplementary Information

Additional file 1: Fig. S1. Clinicopathological features of the TCGA, SWCH, and CGGA cohorts.

Additional file 2: Fig. S2. Relationship between CRGs and clinicopathological features in the TCGA cohort. A Heatmap of the expression of 19 CRGs and clinical parameters. B Relationship between the expression of 19 CRGs and the survival outcomes. C-G Kaplan–Meier curves showing significant differences in survival among five potential key CRGs. *p < 0.05; **p < 0.01; ***p < 0.001.

Additional file 3: Fig. S3. Functional Annotation in distinct risk subgroups. A Venn diagram showing intersecting DEGs between the CRG and immune risk subgroups. B Heatmap for the top 30 upregulated and downregulated genes. C The intersecting genes among the top 30 upregulated and downregulated DEGs in the CRG and immune risk groups. Heatmap of top 15 signaling pathways in the KEGG (D) and HALLMARK (E) gene sets.

Additional file 4: Fig. S4. Immune cell profiles in gliomas. Bar plot (A) and percentage abundance (B) of tumor-infiltrating immune cells showing the distribution of 22 immune cells. C Heatmap illustrating the relationships among CRG risk subgroups, clinical profiles, and 22 types of immune cells.

Additional file 5: Fig. S5. Analysis of tumor mutation, TIDE, immune checkpoints of tumor signature genes in gliomas. A A scatter plot showing TMB was positively correlated with the CRG score. B The characteristics of the top 10 most frequently mutated genes and variant classification. Scatter plot showing the correlation of TIDE, dysfunction, exclusion, and MSI with immune (C-F) and CRG (G-J) scores. K Analysis of immune checkpoints between CRG risk subgroups. L Heatmap illustrating the relationships among CRG risk subgroups, clinical profiles, and 22 types of immune cells. *p < 0.05;**p < 0.01;***p < 0.001*.*

Additional file 6: Fig. S6. Analysis of cytokines in gliomas. A The expression of most cytokines was preferentially detected in patients with high CRG scores. B The majority of 91 cytokines were associated with the CRG score than with the immune score. *p < 0.05;**p < 0.01; ***p < 0.001.

## Data Availability

No datasets were generated or analysed during the current study.

## Abbreviations

CRGs: Cuproptosis-related genes
GBM: Glioblastoma
ICIs: Immune checkpoint inhibitors
CTLA4: Cytotoxic T lymphocyte-associated antigen 4
PD1: Programmed cell death protein 1
TME: Tumor microenvironment
TCGA: The Cancer Genome Atlas
CGGA: Chinese Glioma Genome Atlas
SWCH: Sichuan West China Hospital
LASSO: Least absolute shrinkage and selection operator
DEGs: Differentially Expressed Genes
FC: Fold Change
GO: Gene Ontology
KEGG: Kyoto Encyclopedia of Genes and Genomes
GSEA: Gene set enrichment analysis
ROC: Receiver operating characteristic
TMB: Tumor Mutant Burden
TIDE: Tumor Immune Dysfunction and Exclusion
CTLs: Cytotoxic T Lymphocytes
