# Supplementary figures and images for: The cuproptosis-related signature predicts the prognosis and immune microenvironments of primary diffuse gliomas: a comprehensive analysis
Source: Hum Genomics. 2024 Jul 2;18:74. doi: 10.1186/s40246-024-00636-2 (PMC11220998; doi:10.1186/s40246-024-00636-2)

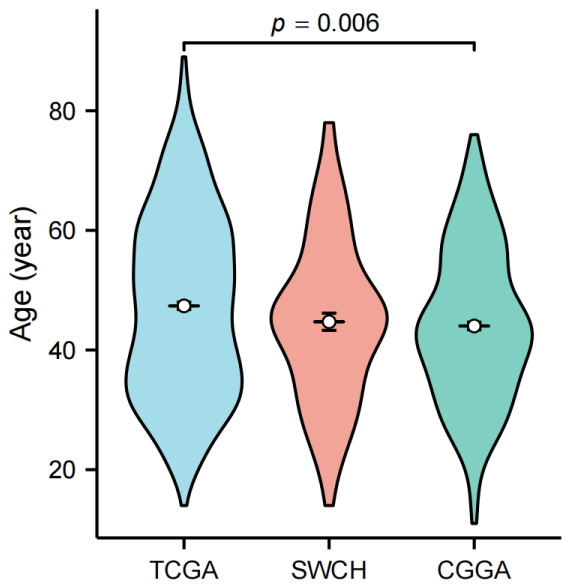

Supplement: Supplementary file 1 — Additional file 1: Fig. S1. Clinicopathological features of the TCGA, SWCH, and CGGA cohorts. [file 40246_2024_636_MOESM1_ESM.zip › Fig. S1A.tif]

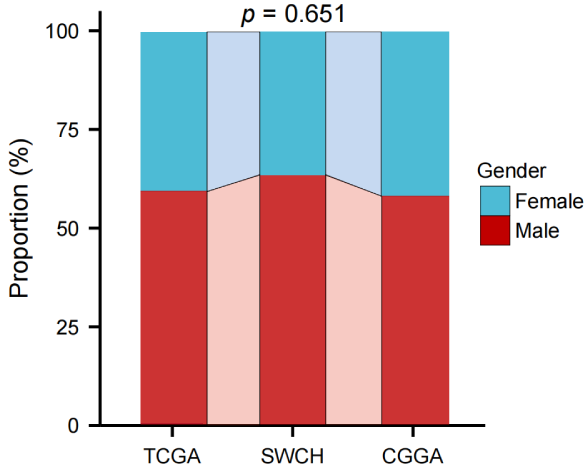

Supplement: Supplementary file 1 — Additional file 1: Fig. S1. Clinicopathological features of the TCGA, SWCH, and CGGA cohorts. [file 40246_2024_636_MOESM1_ESM.zip › Fig. S1B.tif]

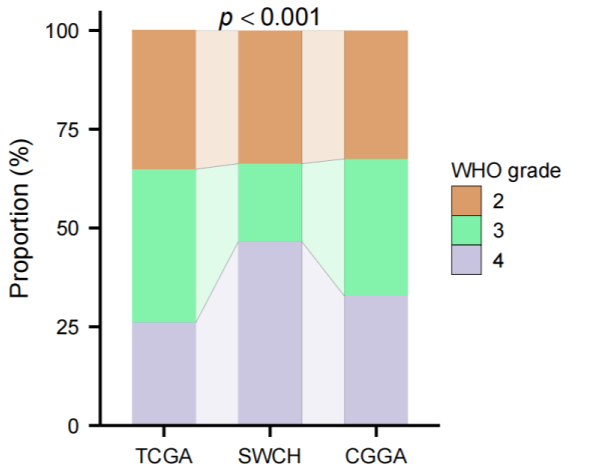

Supplement: Supplementary file 1 — Additional file 1: Fig. S1. Clinicopathological features of the TCGA, SWCH, and CGGA cohorts. [file 40246_2024_636_MOESM1_ESM.zip › Fig. S1C.tif]

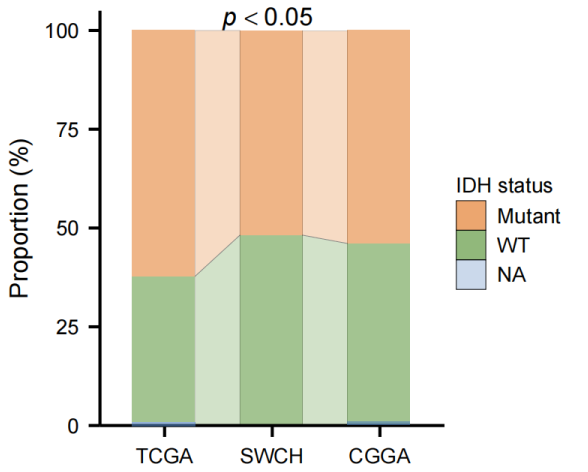

Supplement: Supplementary file 1 — Additional file 1: Fig. S1. Clinicopathological features of the TCGA, SWCH, and CGGA cohorts. [file 40246_2024_636_MOESM1_ESM.zip › Fig. S1D.tif]

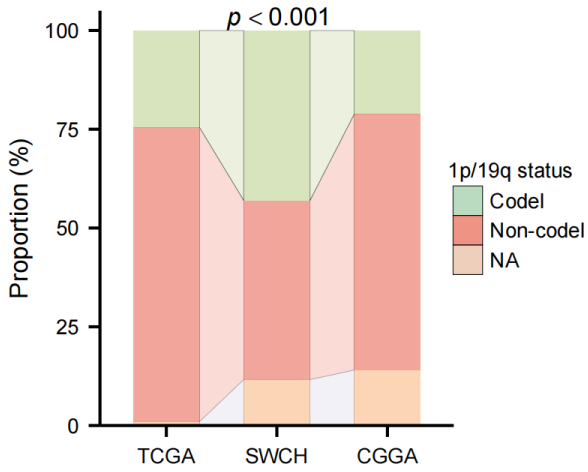

Supplement: Supplementary file 1 — Additional file 1: Fig. S1. Clinicopathological features of the TCGA, SWCH, and CGGA cohorts. [file 40246_2024_636_MOESM1_ESM.zip › Fig. S1E.tif]

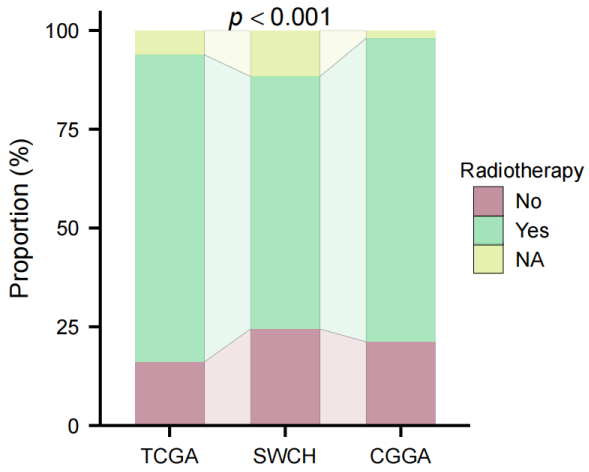

Supplement: Supplementary file 1 — Additional file 1: Fig. S1. Clinicopathological features of the TCGA, SWCH, and CGGA cohorts. [file 40246_2024_636_MOESM1_ESM.zip › Fig. S1F.tif]

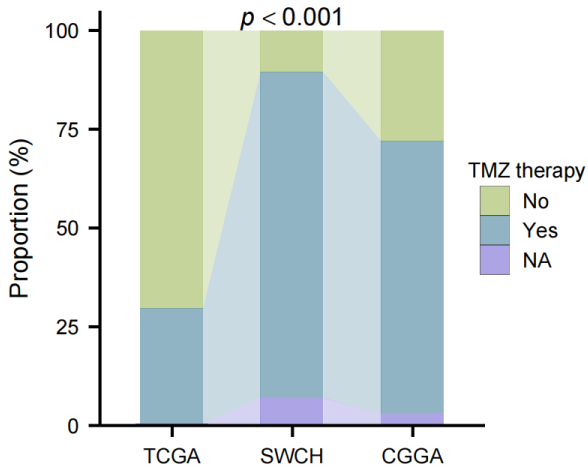

Supplement: Supplementary file 1 — Additional file 1: Fig. S1. Clinicopathological features of the TCGA, SWCH, and CGGA cohorts. [file 40246_2024_636_MOESM1_ESM.zip › Fig. S1G.tif]

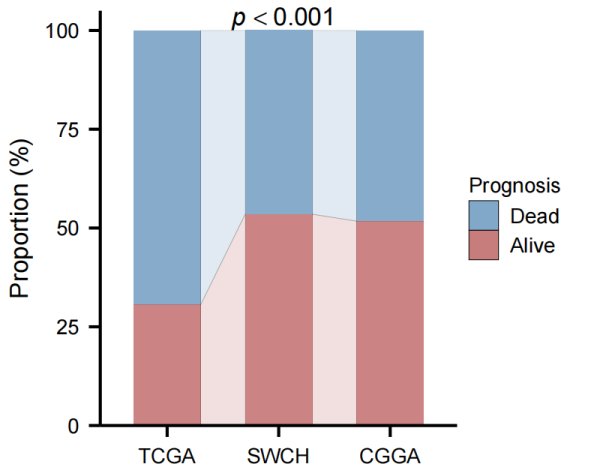

Supplement: Supplementary file 1 — Additional file 1: Fig. S1. Clinicopathological features of the TCGA, SWCH, and CGGA cohorts. [file 40246_2024_636_MOESM1_ESM.zip › Fig. S1H.tif]

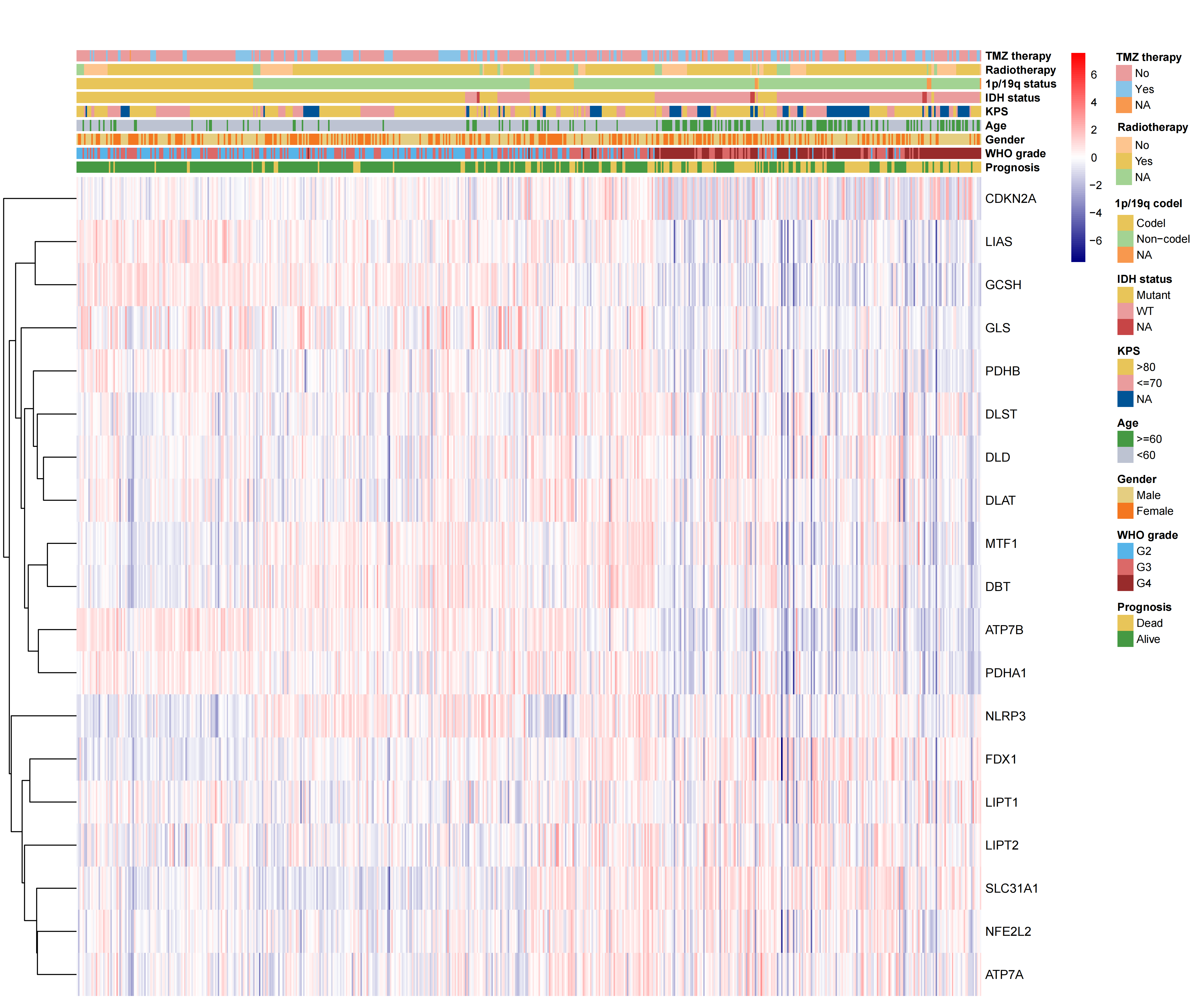

Supplement: Supplementary file 2 — Additional file 2: Fig. S2. Relationship between CRGs and clinicopathological features in the TCGA cohort. A Heatmap of the expression of 19 CRGs and clinical parameters. B Relationship between the expression of 19 CRGs and the survival outcomes. C-G Kaplan–Meier curves showing significant differences in survival among five potential key CRGs. *p < 0.05; **p < 0.01; ***p < 0.001. [file 40246_2024_636_MOESM2_ESM.zip › Fig. S2A.tif]

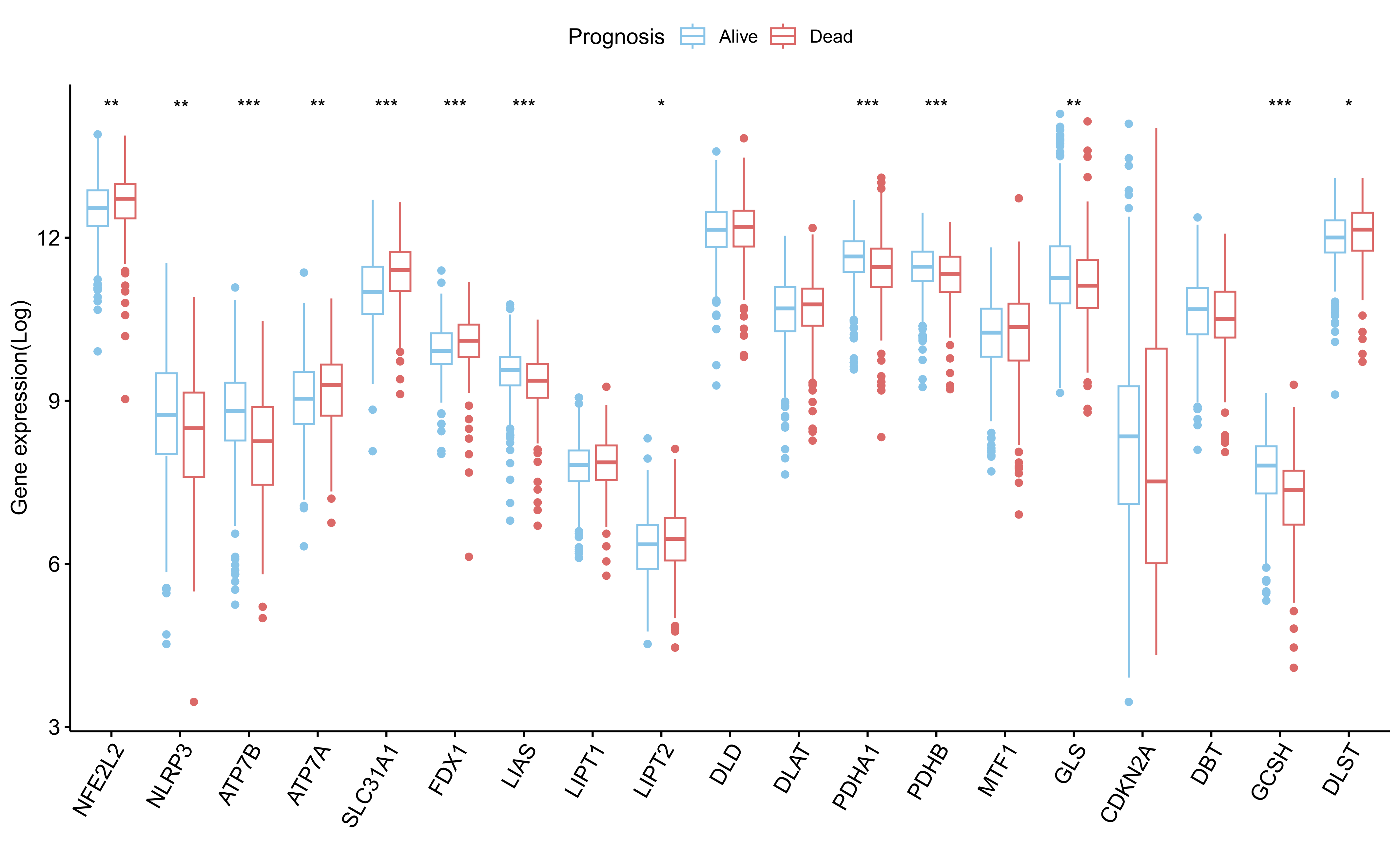

Supplement: Supplementary file 2 — Additional file 2: Fig. S2. Relationship between CRGs and clinicopathological features in the TCGA cohort. A Heatmap of the expression of 19 CRGs and clinical parameters. B Relationship between the expression of 19 CRGs and the survival outcomes. C-G Kaplan–Meier curves showing significant differences in survival among five potential key CRGs. *p < 0.05; **p < 0.01; ***p < 0.001. [file 40246_2024_636_MOESM2_ESM.zip › Fig. S2B.tif]

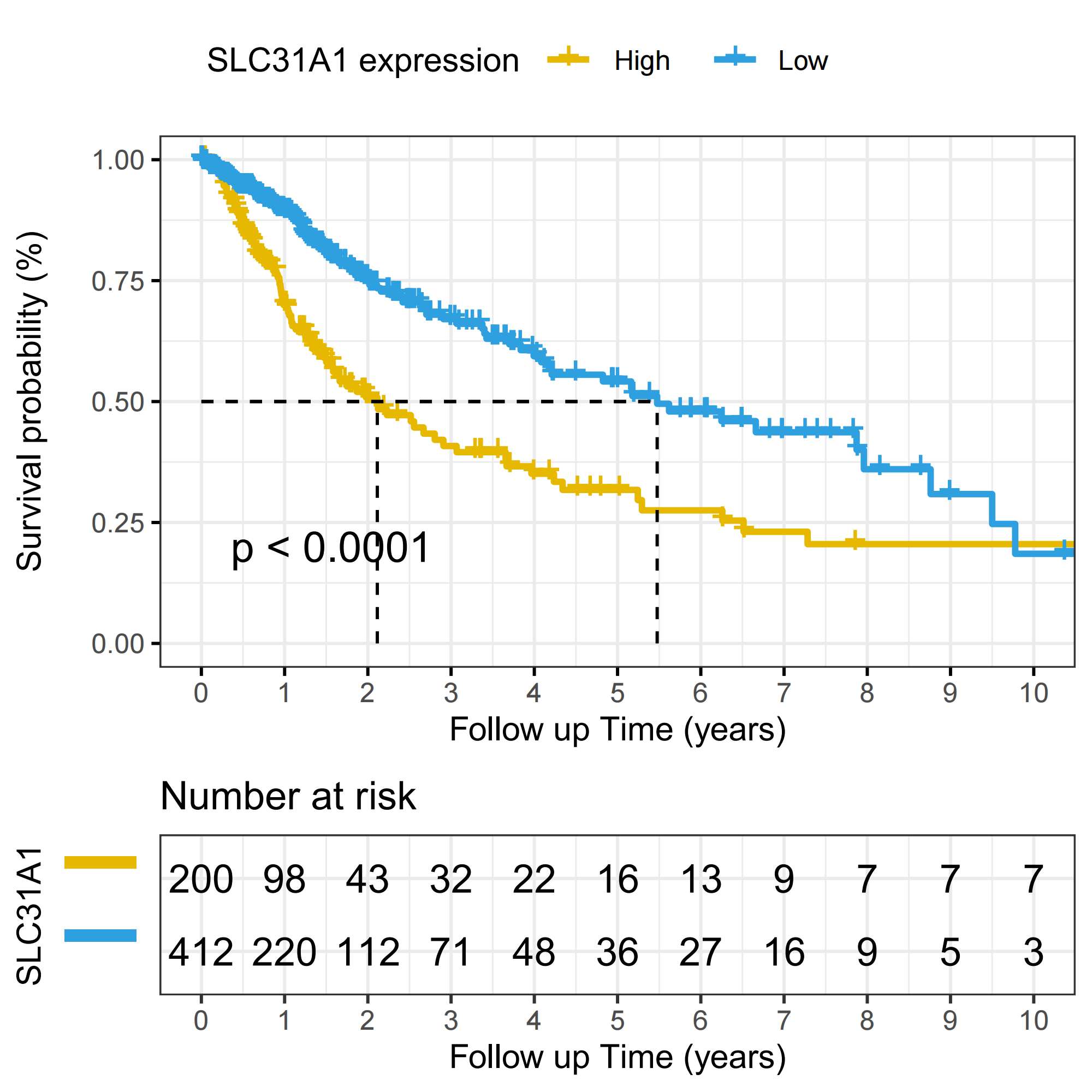

Supplement: Supplementary file 2 — Additional file 2: Fig. S2. Relationship between CRGs and clinicopathological features in the TCGA cohort. A Heatmap of the expression of 19 CRGs and clinical parameters. B Relationship between the expression of 19 CRGs and the survival outcomes. C-G Kaplan–Meier curves showing significant differences in survival among five potential key CRGs. *p < 0.05; **p < 0.01; ***p < 0.001. [file 40246_2024_636_MOESM2_ESM.zip › Fig. S2C.tif]

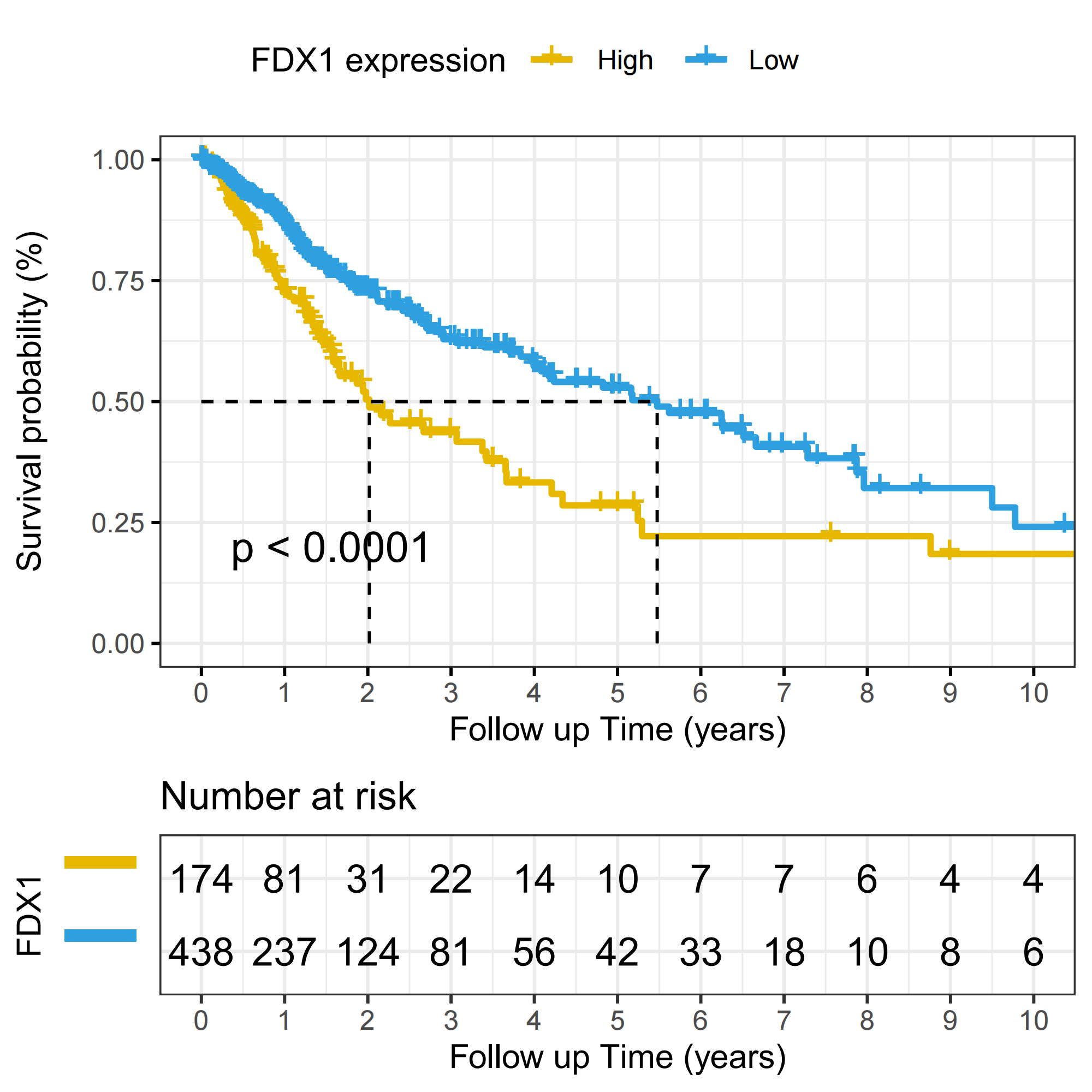

Supplement: Supplementary file 2 — Additional file 2: Fig. S2. Relationship between CRGs and clinicopathological features in the TCGA cohort. A Heatmap of the expression of 19 CRGs and clinical parameters. B Relationship between the expression of 19 CRGs and the survival outcomes. C-G Kaplan–Meier curves showing significant differences in survival among five potential key CRGs. *p < 0.05; **p < 0.01; ***p < 0.001. [file 40246_2024_636_MOESM2_ESM.zip › Fig. S2D.tif]

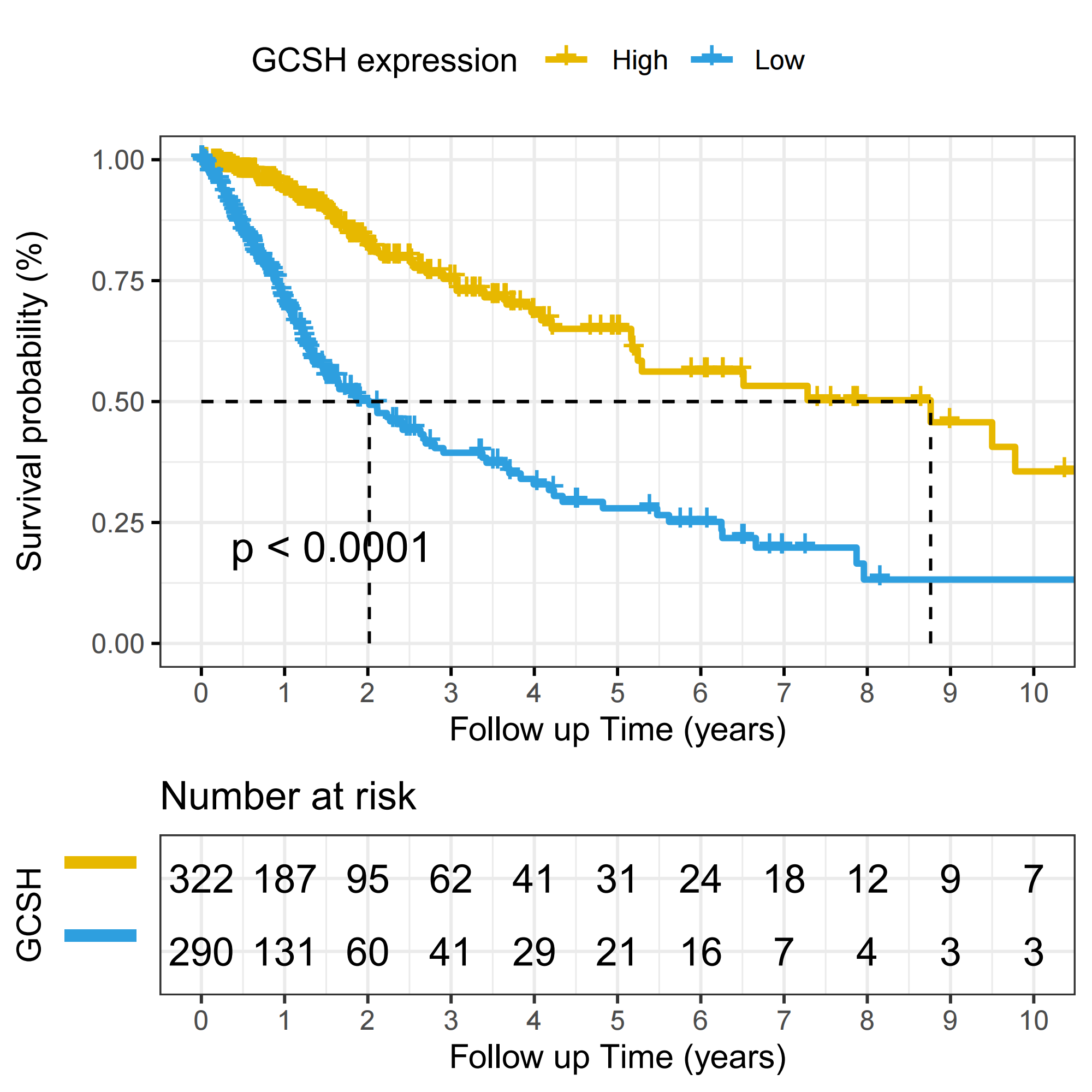

Supplement: Supplementary file 2 — Additional file 2: Fig. S2. Relationship between CRGs and clinicopathological features in the TCGA cohort. A Heatmap of the expression of 19 CRGs and clinical parameters. B Relationship between the expression of 19 CRGs and the survival outcomes. C-G Kaplan–Meier curves showing significant differences in survival among five potential key CRGs. *p < 0.05; **p < 0.01; ***p < 0.001. [file 40246_2024_636_MOESM2_ESM.zip › Fig. S2E.tif]

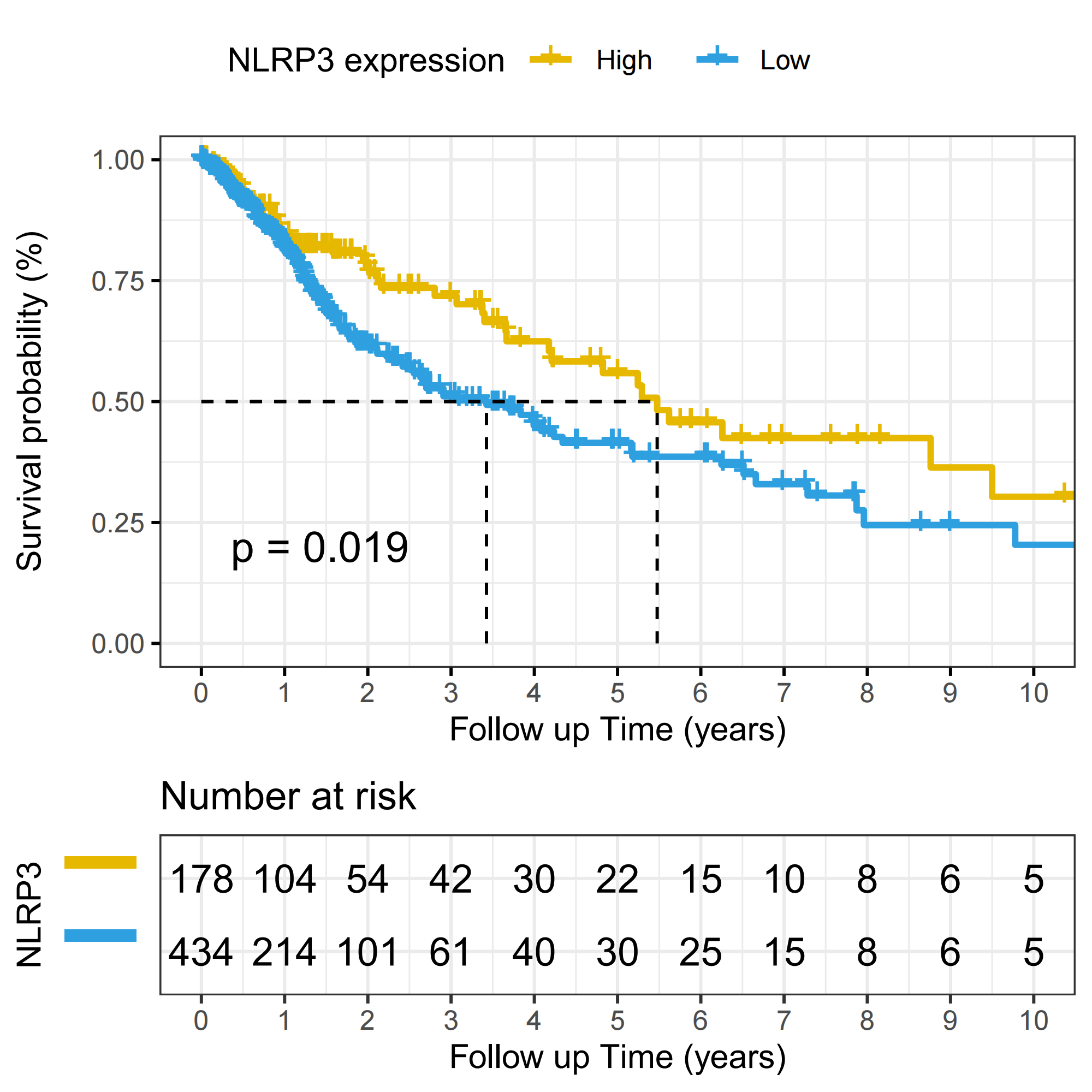

Supplement: Supplementary file 2 — Additional file 2: Fig. S2. Relationship between CRGs and clinicopathological features in the TCGA cohort. A Heatmap of the expression of 19 CRGs and clinical parameters. B Relationship between the expression of 19 CRGs and the survival outcomes. C-G Kaplan–Meier curves showing significant differences in survival among five potential key CRGs. *p < 0.05; **p < 0.01; ***p < 0.001. [file 40246_2024_636_MOESM2_ESM.zip › Fig. S2F.tif]

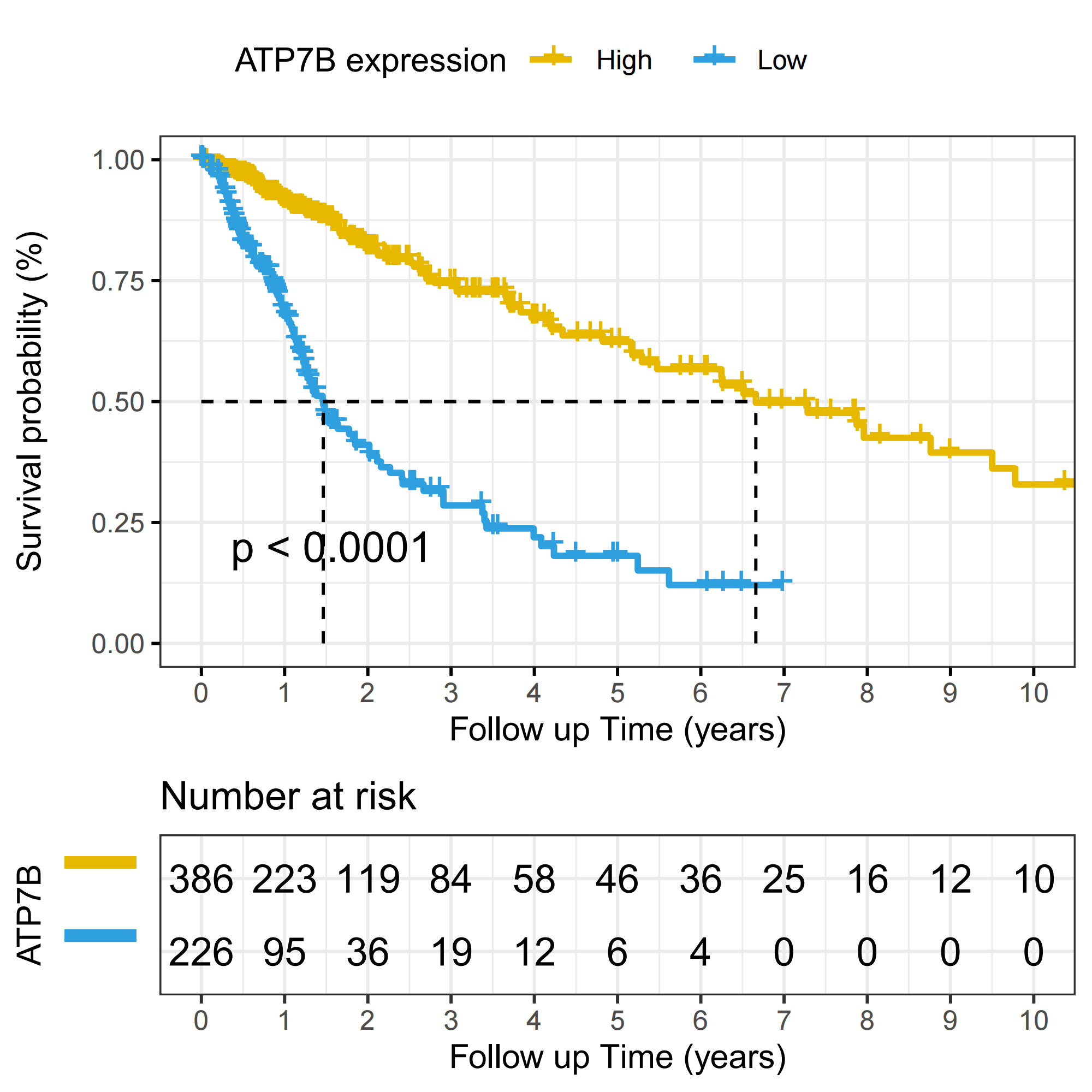

Supplement: Supplementary file 2 — Additional file 2: Fig. S2. Relationship between CRGs and clinicopathological features in the TCGA cohort. A Heatmap of the expression of 19 CRGs and clinical parameters. B Relationship between the expression of 19 CRGs and the survival outcomes. C-G Kaplan–Meier curves showing significant differences in survival among five potential key CRGs. *p < 0.05; **p < 0.01; ***p < 0.001. [file 40246_2024_636_MOESM2_ESM.zip › Fig. S2G.tif]

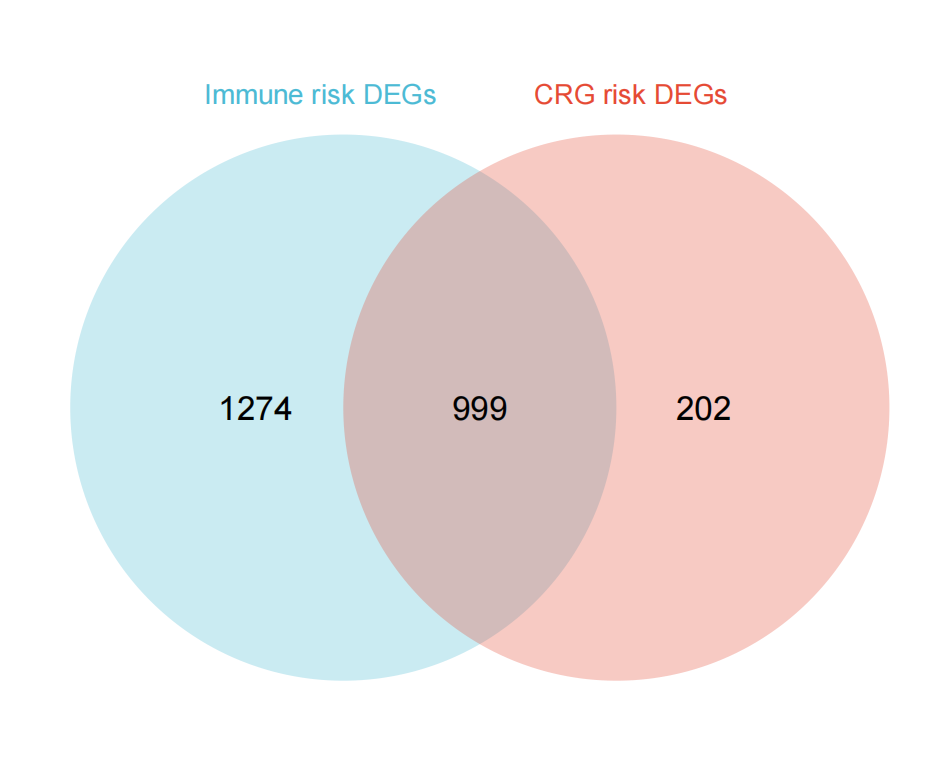

Supplement: Supplementary file 3 — Additional file 3: Fig. S3. Functional Annotation in distinct risk subgroups. A Venn diagram showing intersecting DEGs between the CRG and immune risk subgroups. B Heatmap for the top 30 upregulated and downregulated genes. C The intersecting genes among the top 30 upregulated and downregulated DEGs in the CRG and immune risk groups. Heatmap of top 15 signaling pathways in the KEGG (D) and HALLMARK (E) gene sets. [file 40246_2024_636_MOESM3_ESM.zip › Fig. S3A.tif]

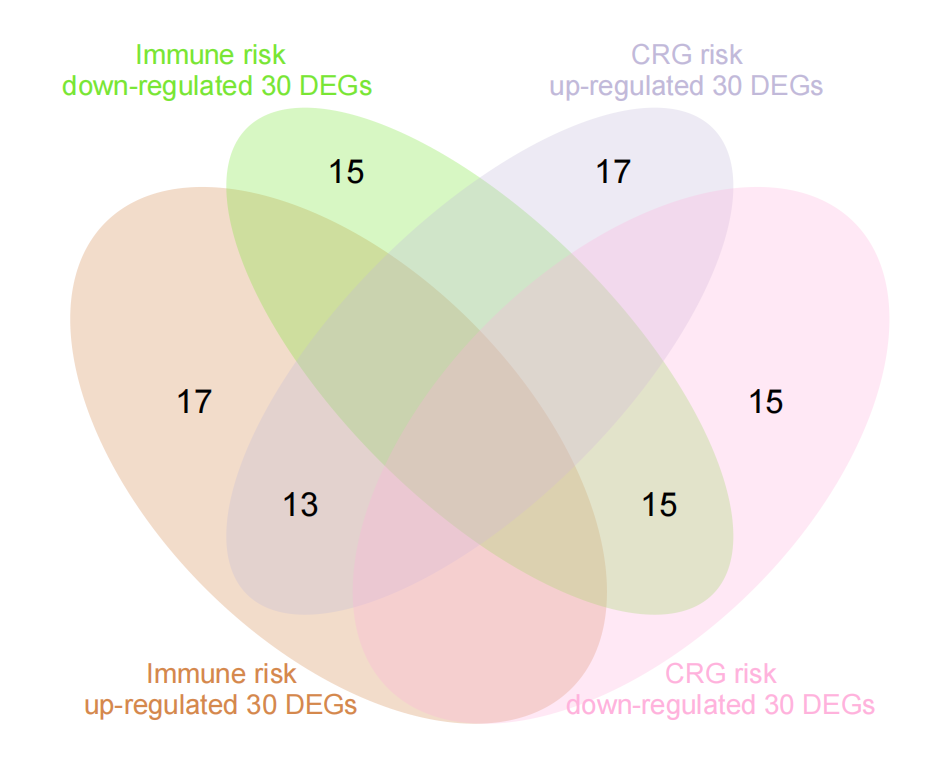

Supplement: Supplementary file 3 — Additional file 3: Fig. S3. Functional Annotation in distinct risk subgroups. A Venn diagram showing intersecting DEGs between the CRG and immune risk subgroups. B Heatmap for the top 30 upregulated and downregulated genes. C The intersecting genes among the top 30 upregulated and downregulated DEGs in the CRG and immune risk groups. Heatmap of top 15 signaling pathways in the KEGG (D) and HALLMARK (E) gene sets. [file 40246_2024_636_MOESM3_ESM.zip › Fig. S3C.tif]

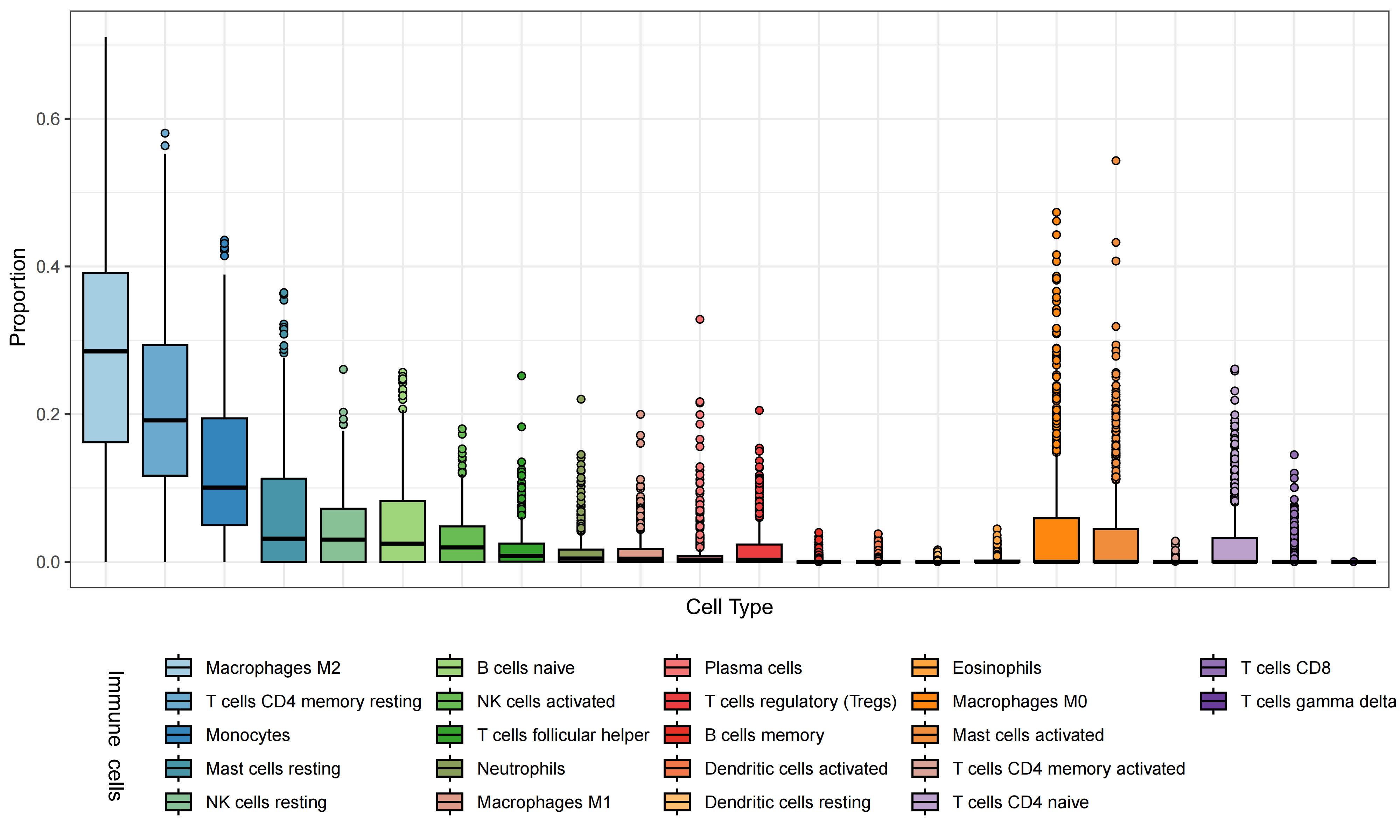

Supplement: Supplementary file 4 — Additional file 4: Fig. S4. Immune cell profiles in gliomas. Bar plot (A) and percentage abundance (B) of tumor-infiltrating immune cells showing the distribution of 22 immune cells. C Heatmap illustrating the relationships among CRG risk subgroups, clinical profiles, and 22 types of immune cells. [file 40246_2024_636_MOESM4_ESM.zip › Fig. S4A.tif]

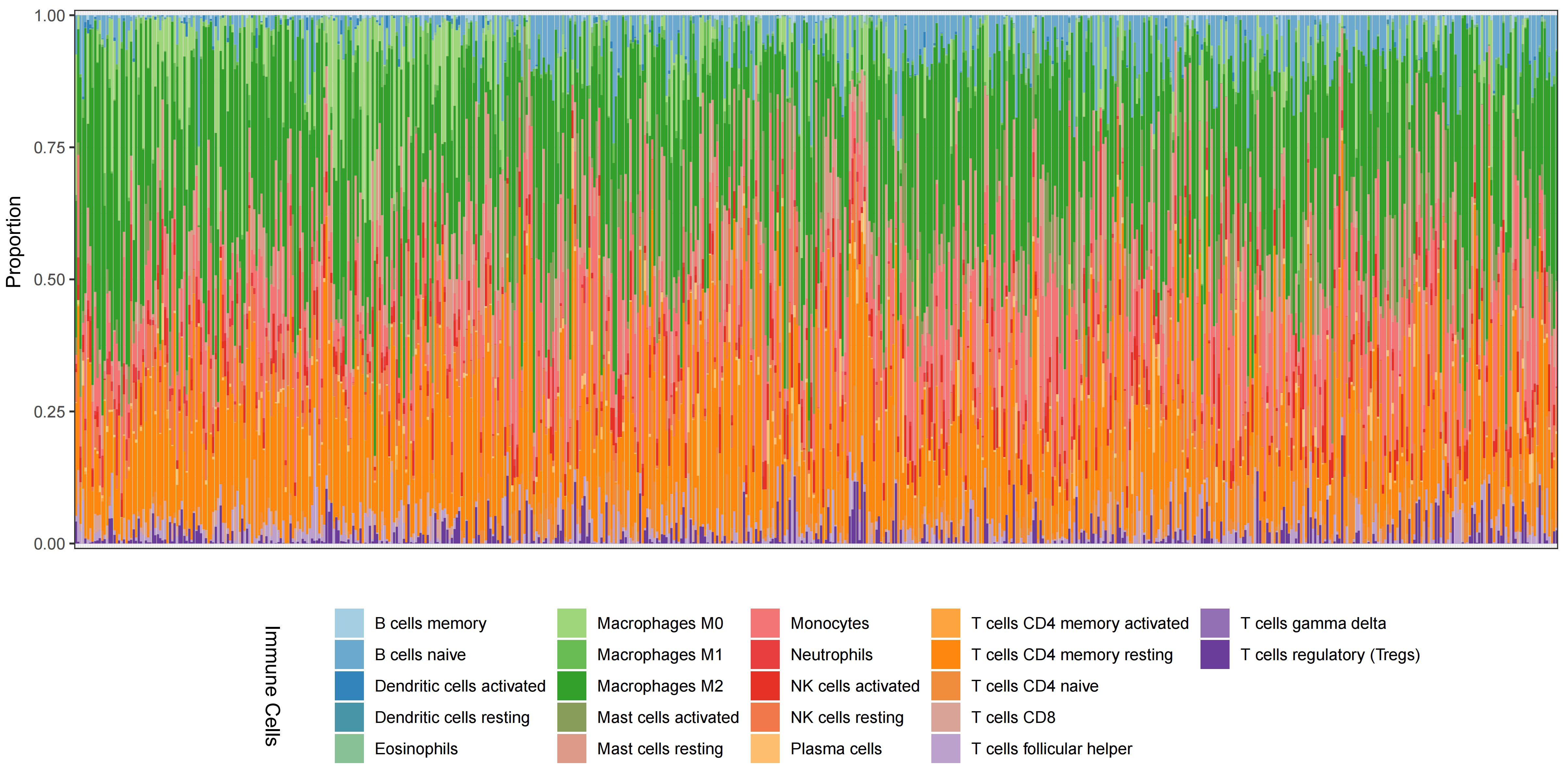

Supplement: Supplementary file 4 — Additional file 4: Fig. S4. Immune cell profiles in gliomas. Bar plot (A) and percentage abundance (B) of tumor-infiltrating immune cells showing the distribution of 22 immune cells. C Heatmap illustrating the relationships among CRG risk subgroups, clinical profiles, and 22 types of immune cells. [file 40246_2024_636_MOESM4_ESM.zip › Fig. S4B.tif]

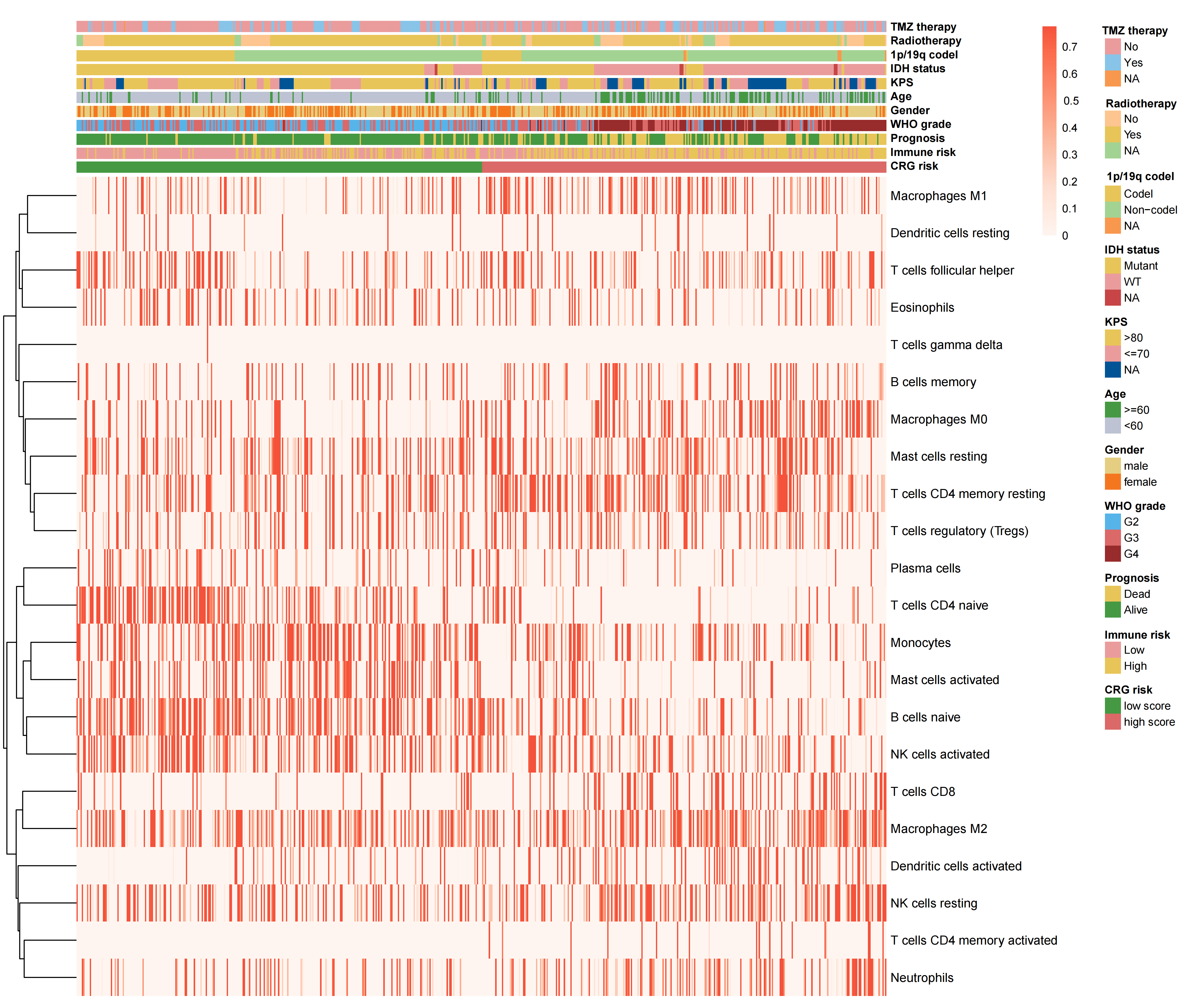

Supplement: Supplementary file 4 — Additional file 4: Fig. S4. Immune cell profiles in gliomas. Bar plot (A) and percentage abundance (B) of tumor-infiltrating immune cells showing the distribution of 22 immune cells. C Heatmap illustrating the relationships among CRG risk subgroups, clinical profiles, and 22 types of immune cells. [file 40246_2024_636_MOESM4_ESM.zip › Fig. S4C.tif]

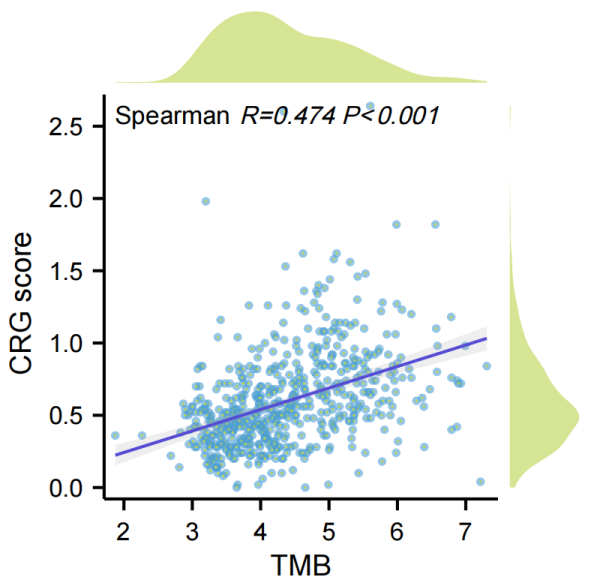

Supplement: Supplementary file 5 — Additional file 5: Fig. S5. Analysis of tumor mutation, TIDE, immune checkpoints of tumor signature genes in gliomas. A A scatter plot showing TMB was positively correlated with the CRG score. B The characteristics of the top 10 most frequently mutated genes and variant classification. Scatter plot showing the correlation of TIDE, dysfunction, exclusion, and MSI with immune (C-F) and CRG (G-J) scores. K Analysis of immune checkpoints between CRG risk subgroups. L Heatmap illustrating the relationships among CRG risk subgroups, clinical profiles, and 22 types of immune cells. *p < 0.05;**p < 0.01;***p < 0.001. [file 40246_2024_636_MOESM5_ESM.zip › Fig. S5A.tif]

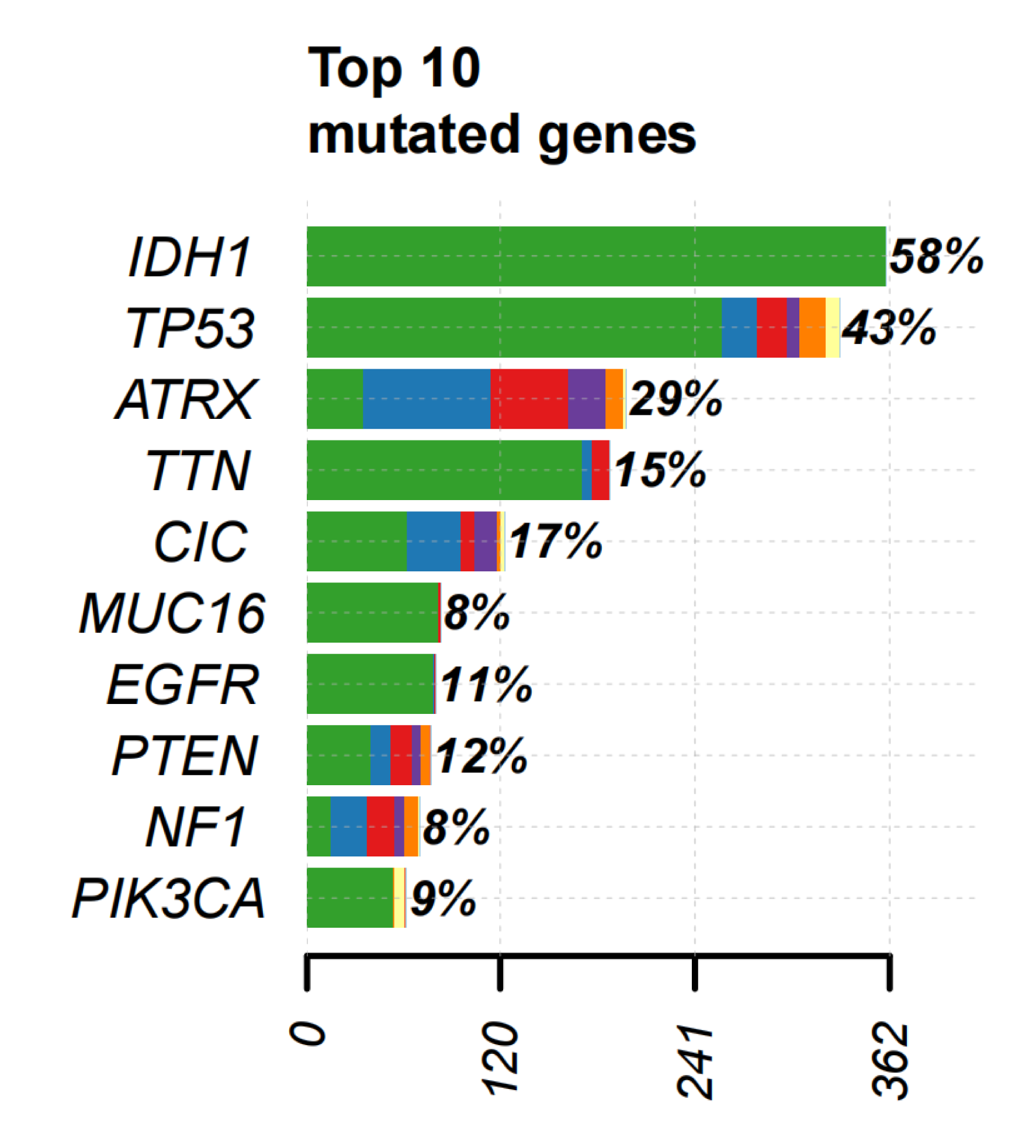

Supplement: Supplementary file 5 — Additional file 5: Fig. S5. Analysis of tumor mutation, TIDE, immune checkpoints of tumor signature genes in gliomas. A A scatter plot showing TMB was positively correlated with the CRG score. B The characteristics of the top 10 most frequently mutated genes and variant classification. Scatter plot showing the correlation of TIDE, dysfunction, exclusion, and MSI with immune (C-F) and CRG (G-J) scores. K Analysis of immune checkpoints between CRG risk subgroups. L Heatmap illustrating the relationships among CRG risk subgroups, clinical profiles, and 22 types of immune cells. *p < 0.05;**p < 0.01;***p < 0.001. [file 40246_2024_636_MOESM5_ESM.zip › Fig. S5B1.tif]

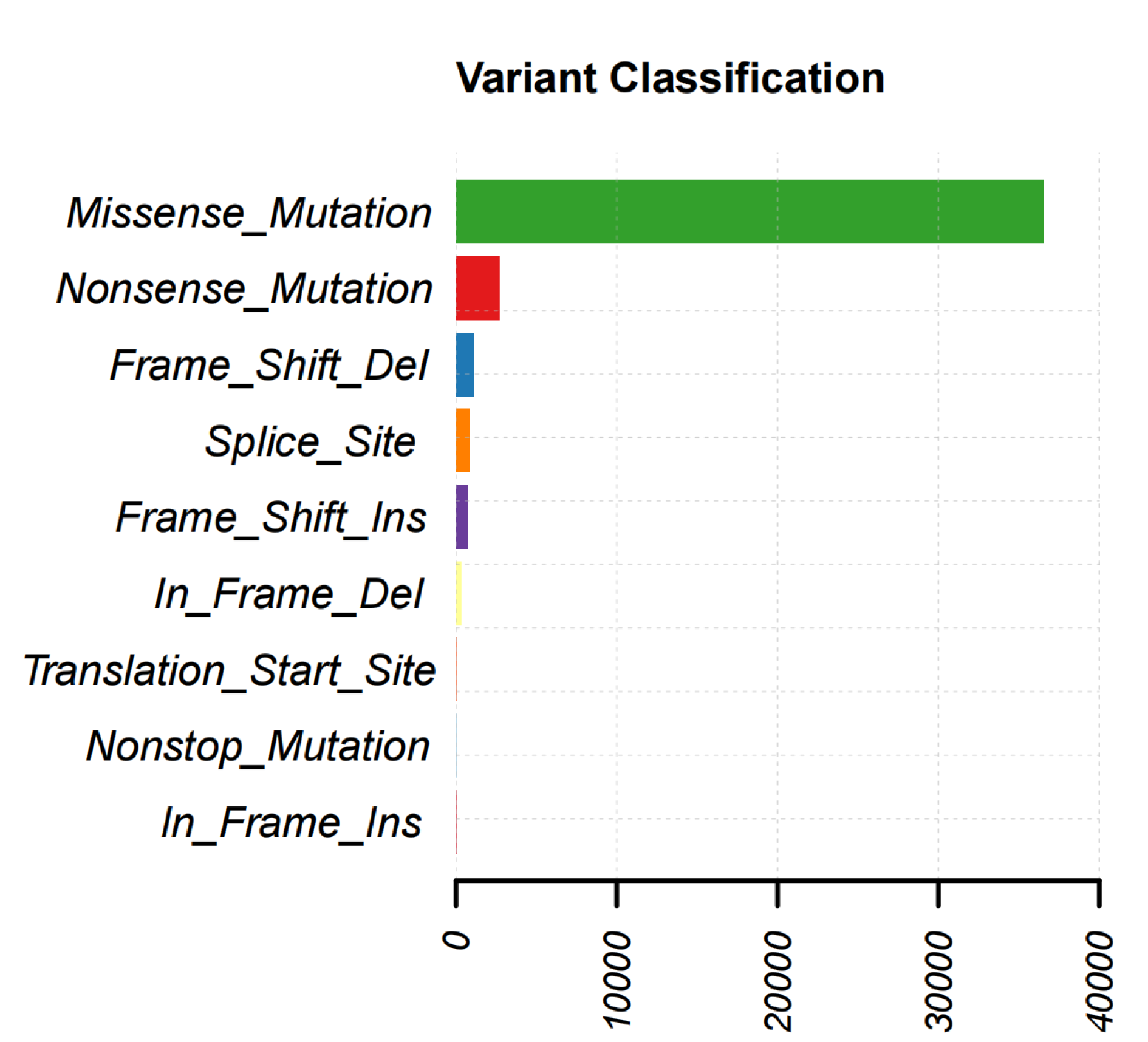

Supplement: Supplementary file 5 — Additional file 5: Fig. S5. Analysis of tumor mutation, TIDE, immune checkpoints of tumor signature genes in gliomas. A A scatter plot showing TMB was positively correlated with the CRG score. B The characteristics of the top 10 most frequently mutated genes and variant classification. Scatter plot showing the correlation of TIDE, dysfunction, exclusion, and MSI with immune (C-F) and CRG (G-J) scores. K Analysis of immune checkpoints between CRG risk subgroups. L Heatmap illustrating the relationships among CRG risk subgroups, clinical profiles, and 22 types of immune cells. *p < 0.05;**p < 0.01;***p < 0.001. [file 40246_2024_636_MOESM5_ESM.zip › Fig. S5B2.tif]

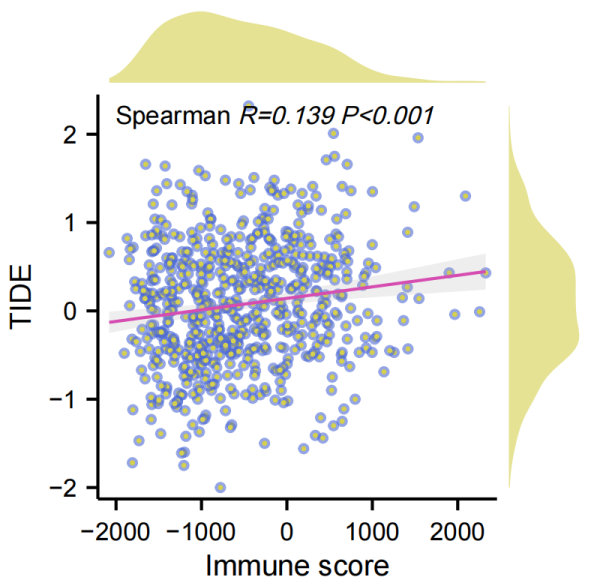

Supplement: Supplementary file 5 — Additional file 5: Fig. S5. Analysis of tumor mutation, TIDE, immune checkpoints of tumor signature genes in gliomas. A A scatter plot showing TMB was positively correlated with the CRG score. B The characteristics of the top 10 most frequently mutated genes and variant classification. Scatter plot showing the correlation of TIDE, dysfunction, exclusion, and MSI with immune (C-F) and CRG (G-J) scores. K Analysis of immune checkpoints between CRG risk subgroups. L Heatmap illustrating the relationships among CRG risk subgroups, clinical profiles, and 22 types of immune cells. *p < 0.05;**p < 0.01;***p < 0.001. [file 40246_2024_636_MOESM5_ESM.zip › Fig. S5C.tif]

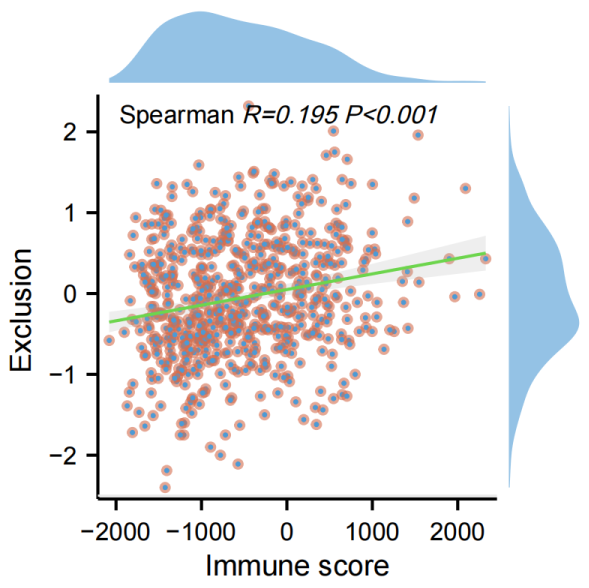

Supplement: Supplementary file 5 — Additional file 5: Fig. S5. Analysis of tumor mutation, TIDE, immune checkpoints of tumor signature genes in gliomas. A A scatter plot showing TMB was positively correlated with the CRG score. B The characteristics of the top 10 most frequently mutated genes and variant classification. Scatter plot showing the correlation of TIDE, dysfunction, exclusion, and MSI with immune (C-F) and CRG (G-J) scores. K Analysis of immune checkpoints between CRG risk subgroups. L Heatmap illustrating the relationships among CRG risk subgroups, clinical profiles, and 22 types of immune cells. *p < 0.05;**p < 0.01;***p < 0.001. [file 40246_2024_636_MOESM5_ESM.zip › Fig. S5D.tif]

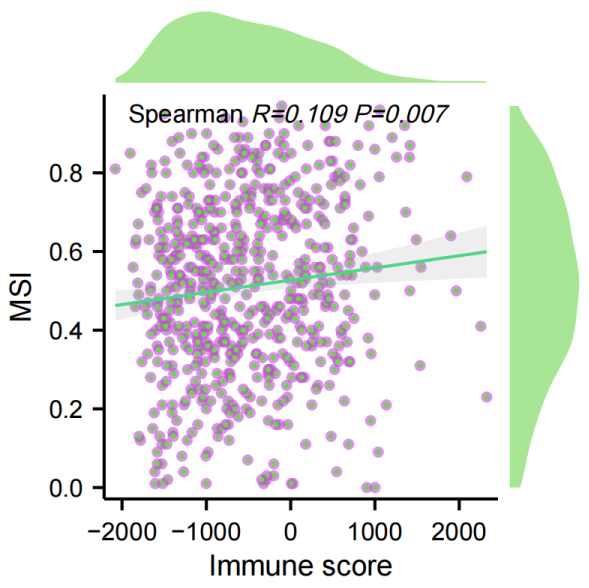

Supplement: Supplementary file 5 — Additional file 5: Fig. S5. Analysis of tumor mutation, TIDE, immune checkpoints of tumor signature genes in gliomas. A A scatter plot showing TMB was positively correlated with the CRG score. B The characteristics of the top 10 most frequently mutated genes and variant classification. Scatter plot showing the correlation of TIDE, dysfunction, exclusion, and MSI with immune (C-F) and CRG (G-J) scores. K Analysis of immune checkpoints between CRG risk subgroups. L Heatmap illustrating the relationships among CRG risk subgroups, clinical profiles, and 22 types of immune cells. *p < 0.05;**p < 0.01;***p < 0.001. [file 40246_2024_636_MOESM5_ESM.zip › Fig. S5E.tif]

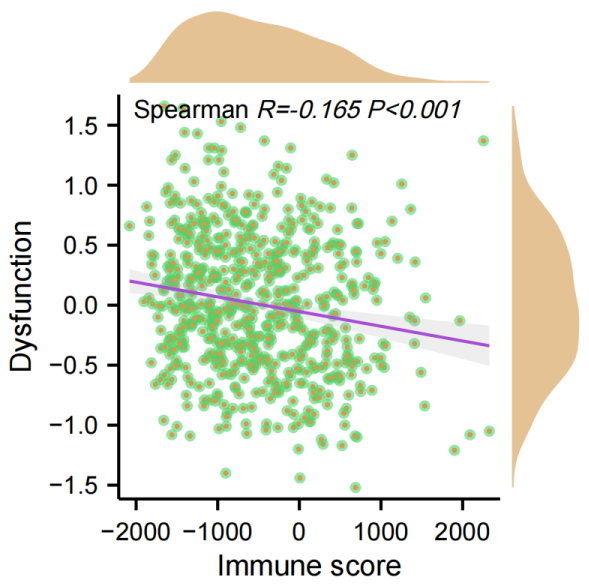

Supplement: Supplementary file 5 — Additional file 5: Fig. S5. Analysis of tumor mutation, TIDE, immune checkpoints of tumor signature genes in gliomas. A A scatter plot showing TMB was positively correlated with the CRG score. B The characteristics of the top 10 most frequently mutated genes and variant classification. Scatter plot showing the correlation of TIDE, dysfunction, exclusion, and MSI with immune (C-F) and CRG (G-J) scores. K Analysis of immune checkpoints between CRG risk subgroups. L Heatmap illustrating the relationships among CRG risk subgroups, clinical profiles, and 22 types of immune cells. *p < 0.05;**p < 0.01;***p < 0.001. [file 40246_2024_636_MOESM5_ESM.zip › Fig. S5F.tif]

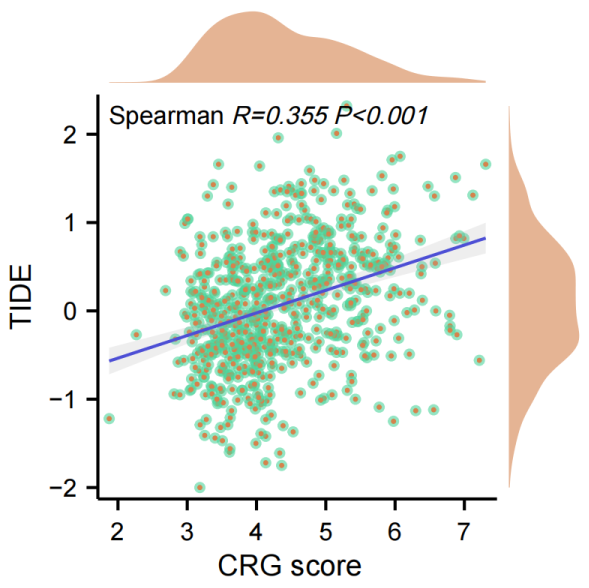

Supplement: Supplementary file 5 — Additional file 5: Fig. S5. Analysis of tumor mutation, TIDE, immune checkpoints of tumor signature genes in gliomas. A A scatter plot showing TMB was positively correlated with the CRG score. B The characteristics of the top 10 most frequently mutated genes and variant classification. Scatter plot showing the correlation of TIDE, dysfunction, exclusion, and MSI with immune (C-F) and CRG (G-J) scores. K Analysis of immune checkpoints between CRG risk subgroups. L Heatmap illustrating the relationships among CRG risk subgroups, clinical profiles, and 22 types of immune cells. *p < 0.05;**p < 0.01;***p < 0.001. [file 40246_2024_636_MOESM5_ESM.zip › Fig. S5G.tif]

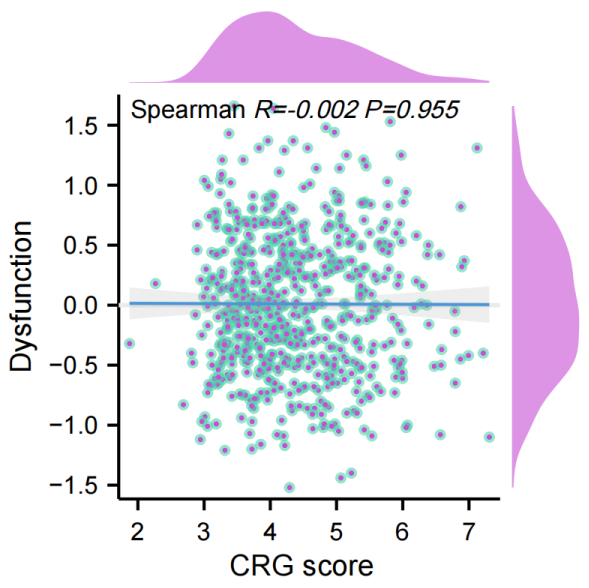

Supplement: Supplementary file 5 — Additional file 5: Fig. S5. Analysis of tumor mutation, TIDE, immune checkpoints of tumor signature genes in gliomas. A A scatter plot showing TMB was positively correlated with the CRG score. B The characteristics of the top 10 most frequently mutated genes and variant classification. Scatter plot showing the correlation of TIDE, dysfunction, exclusion, and MSI with immune (C-F) and CRG (G-J) scores. K Analysis of immune checkpoints between CRG risk subgroups. L Heatmap illustrating the relationships among CRG risk subgroups, clinical profiles, and 22 types of immune cells. *p < 0.05;**p < 0.01;***p < 0.001. [file 40246_2024_636_MOESM5_ESM.zip › Fig. S5H.tif]

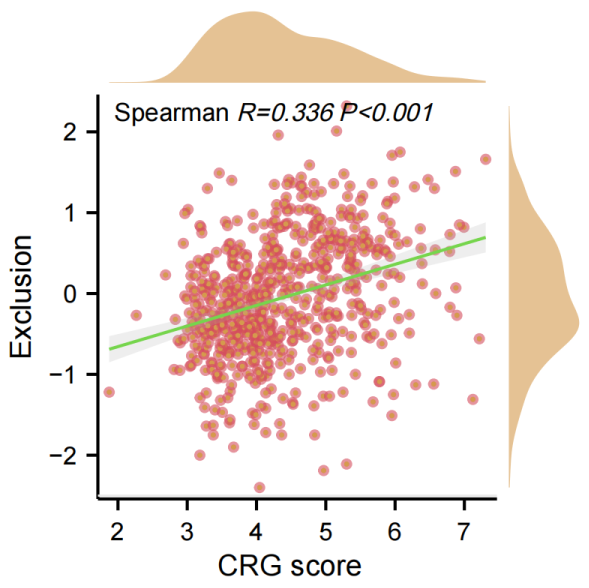

Supplement: Supplementary file 5 — Additional file 5: Fig. S5. Analysis of tumor mutation, TIDE, immune checkpoints of tumor signature genes in gliomas. A A scatter plot showing TMB was positively correlated with the CRG score. B The characteristics of the top 10 most frequently mutated genes and variant classification. Scatter plot showing the correlation of TIDE, dysfunction, exclusion, and MSI with immune (C-F) and CRG (G-J) scores. K Analysis of immune checkpoints between CRG risk subgroups. L Heatmap illustrating the relationships among CRG risk subgroups, clinical profiles, and 22 types of immune cells. *p < 0.05;**p < 0.01;***p < 0.001. [file 40246_2024_636_MOESM5_ESM.zip › Fig. S5I.tif]

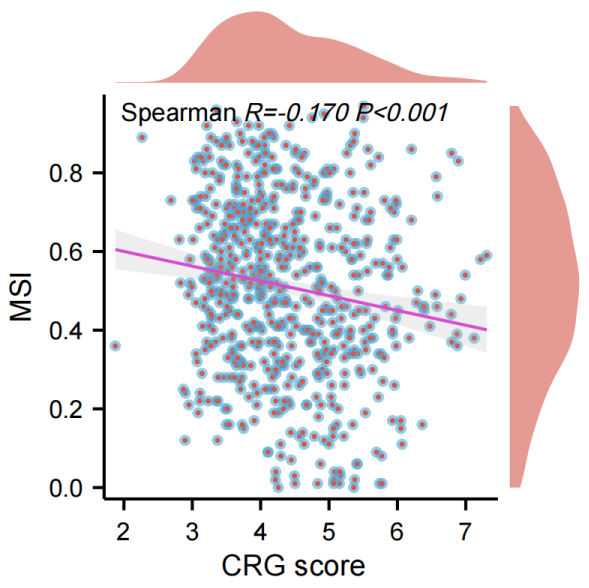

Supplement: Supplementary file 5 — Additional file 5: Fig. S5. Analysis of tumor mutation, TIDE, immune checkpoints of tumor signature genes in gliomas. A A scatter plot showing TMB was positively correlated with the CRG score. B The characteristics of the top 10 most frequently mutated genes and variant classification. Scatter plot showing the correlation of TIDE, dysfunction, exclusion, and MSI with immune (C-F) and CRG (G-J) scores. K Analysis of immune checkpoints between CRG risk subgroups. L Heatmap illustrating the relationships among CRG risk subgroups, clinical profiles, and 22 types of immune cells. *p < 0.05;**p < 0.01;***p < 0.001. [file 40246_2024_636_MOESM5_ESM.zip › Fig. S5J.tif]

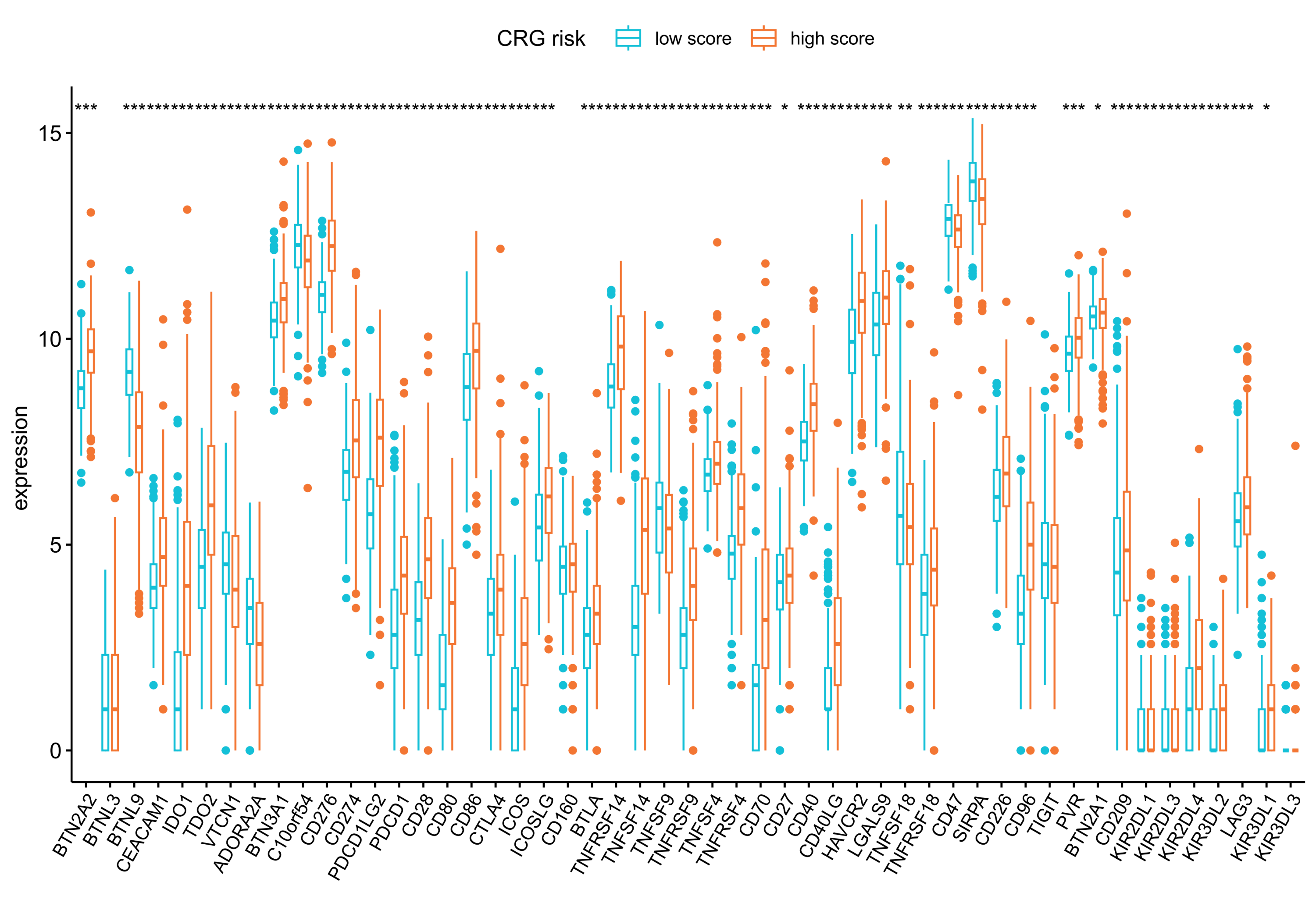

Supplement: Supplementary file 5 — Additional file 5: Fig. S5. Analysis of tumor mutation, TIDE, immune checkpoints of tumor signature genes in gliomas. A A scatter plot showing TMB was positively correlated with the CRG score. B The characteristics of the top 10 most frequently mutated genes and variant classification. Scatter plot showing the correlation of TIDE, dysfunction, exclusion, and MSI with immune (C-F) and CRG (G-J) scores. K Analysis of immune checkpoints between CRG risk subgroups. L Heatmap illustrating the relationships among CRG risk subgroups, clinical profiles, and 22 types of immune cells. *p < 0.05;**p < 0.01;***p < 0.001. [file 40246_2024_636_MOESM5_ESM.zip › Fig. S5K.tif]

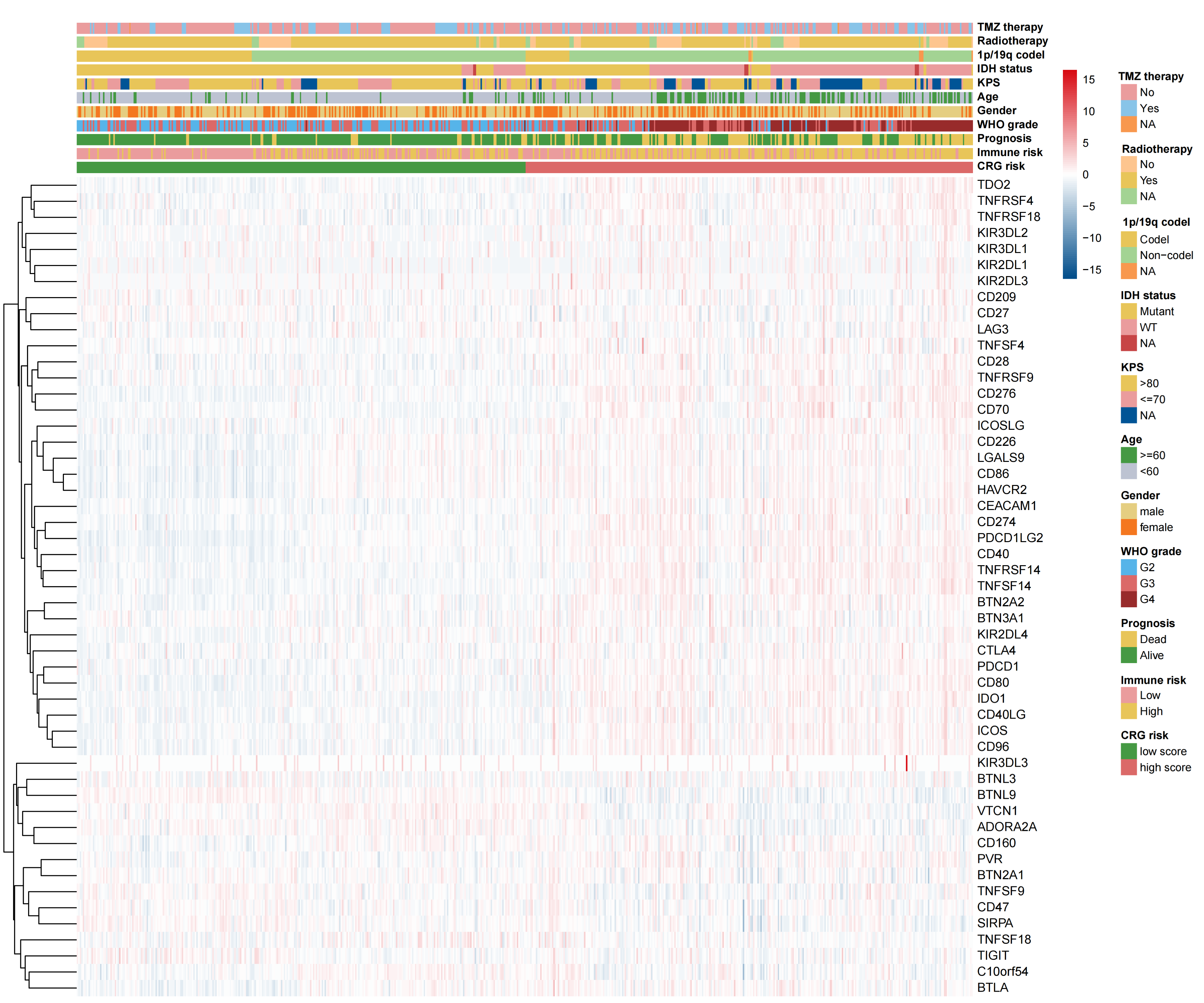

Supplement: Supplementary file 5 — Additional file 5: Fig. S5. Analysis of tumor mutation, TIDE, immune checkpoints of tumor signature genes in gliomas. A A scatter plot showing TMB was positively correlated with the CRG score. B The characteristics of the top 10 most frequently mutated genes and variant classification. Scatter plot showing the correlation of TIDE, dysfunction, exclusion, and MSI with immune (C-F) and CRG (G-J) scores. K Analysis of immune checkpoints between CRG risk subgroups. L Heatmap illustrating the relationships among CRG risk subgroups, clinical profiles, and 22 types of immune cells. *p < 0.05;**p < 0.01;***p < 0.001. [file 40246_2024_636_MOESM5_ESM.zip › Fig. S5L.tif]

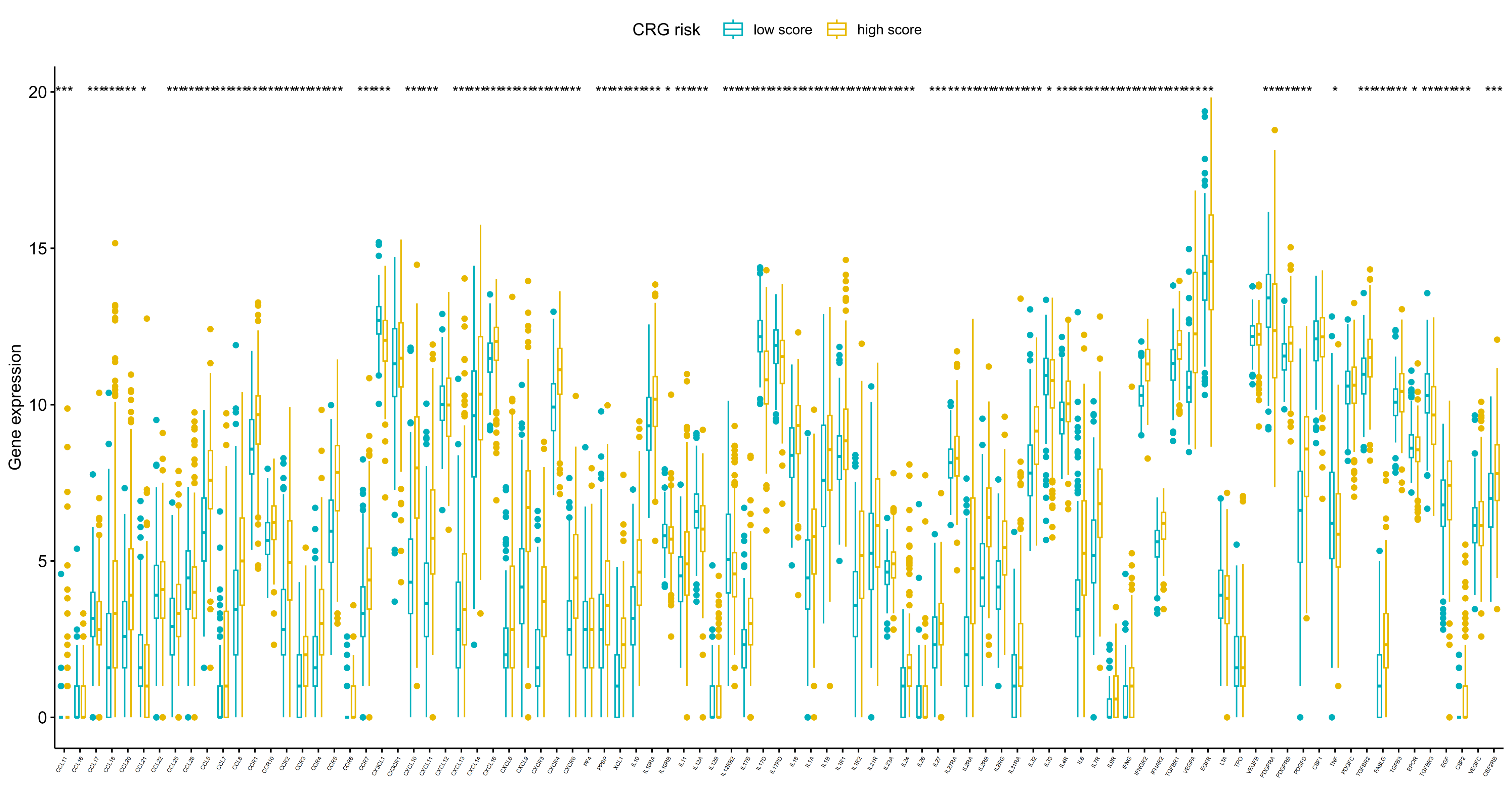

Supplement: Supplementary file 6 — Additional file 6: Fig. S6. Analysis of cytokines in gliomas. A The expression of most cytokines was preferentially detected in patients with high CRG scores. B The majority of 91 cytokines were associated with the CRG score than with the immune score. *p < 0.05;**p < 0.01; ***p < 0.001. [file 40246_2024_636_MOESM6_ESM.zip › Fig. S6A.tif]

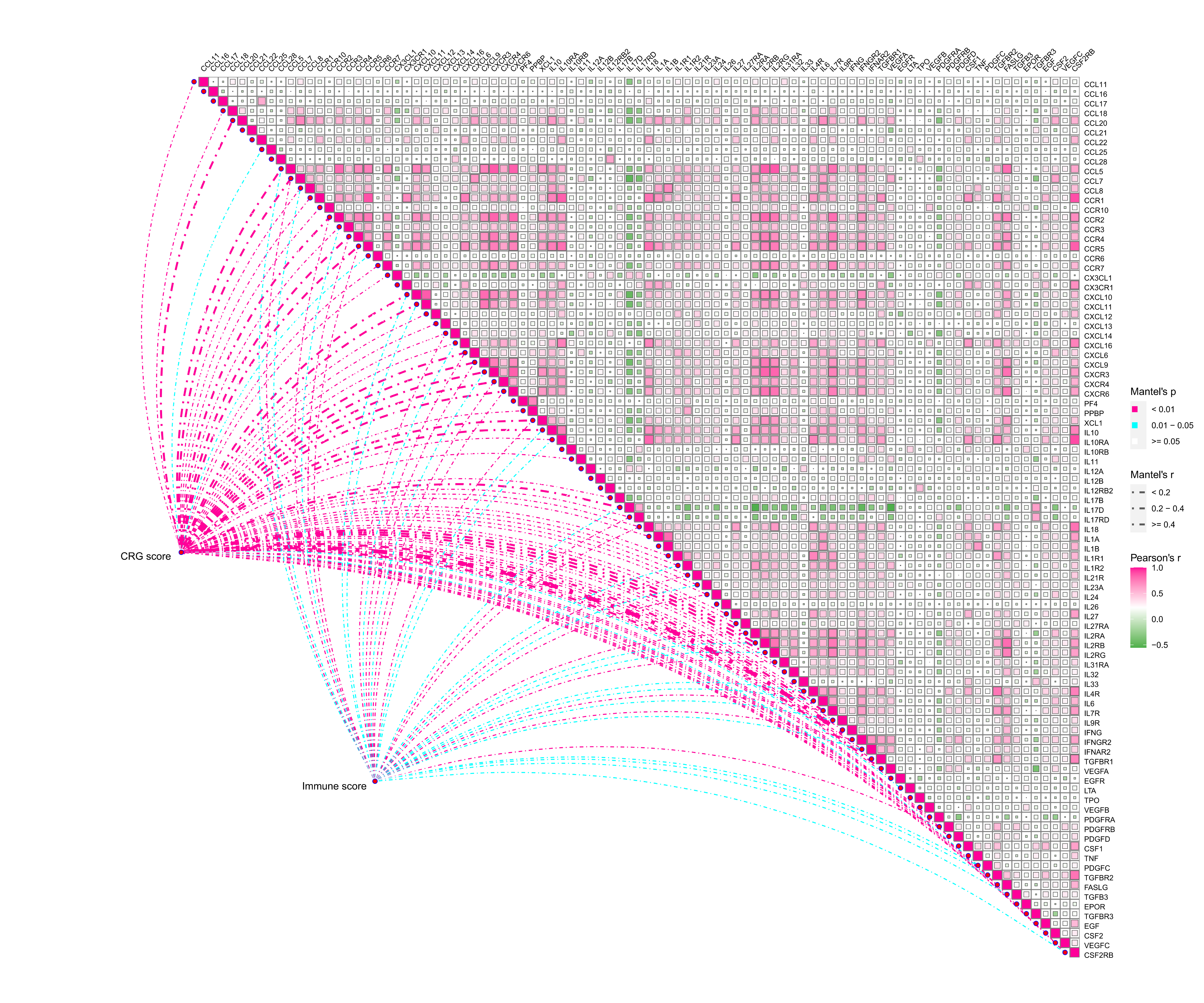

Supplement: Supplementary file 6 — Additional file 6: Fig. S6. Analysis of cytokines in gliomas. A The expression of most cytokines was preferentially detected in patients with high CRG scores. B The majority of 91 cytokines were associated with the CRG score than with the immune score. *p < 0.05;**p < 0.01; ***p < 0.001. [file 40246_2024_636_MOESM6_ESM.zip › Fig. S6B.tif]
